# Supplementary material for: An ARF GTPase module promoting invasion and metastasis through regulating phosphoinositide metabolism
Source: Nat Commun. 2021 Mar 12;12:1623. doi: 10.1038/s41467-021-21847-4 (PMC7955138; doi:10.1038/s41467-021-21847-4)
Supplement: Supplementary file 1 — Supplementary Information [file 41467_2021_21847_MOESM1_ESM.pdf]

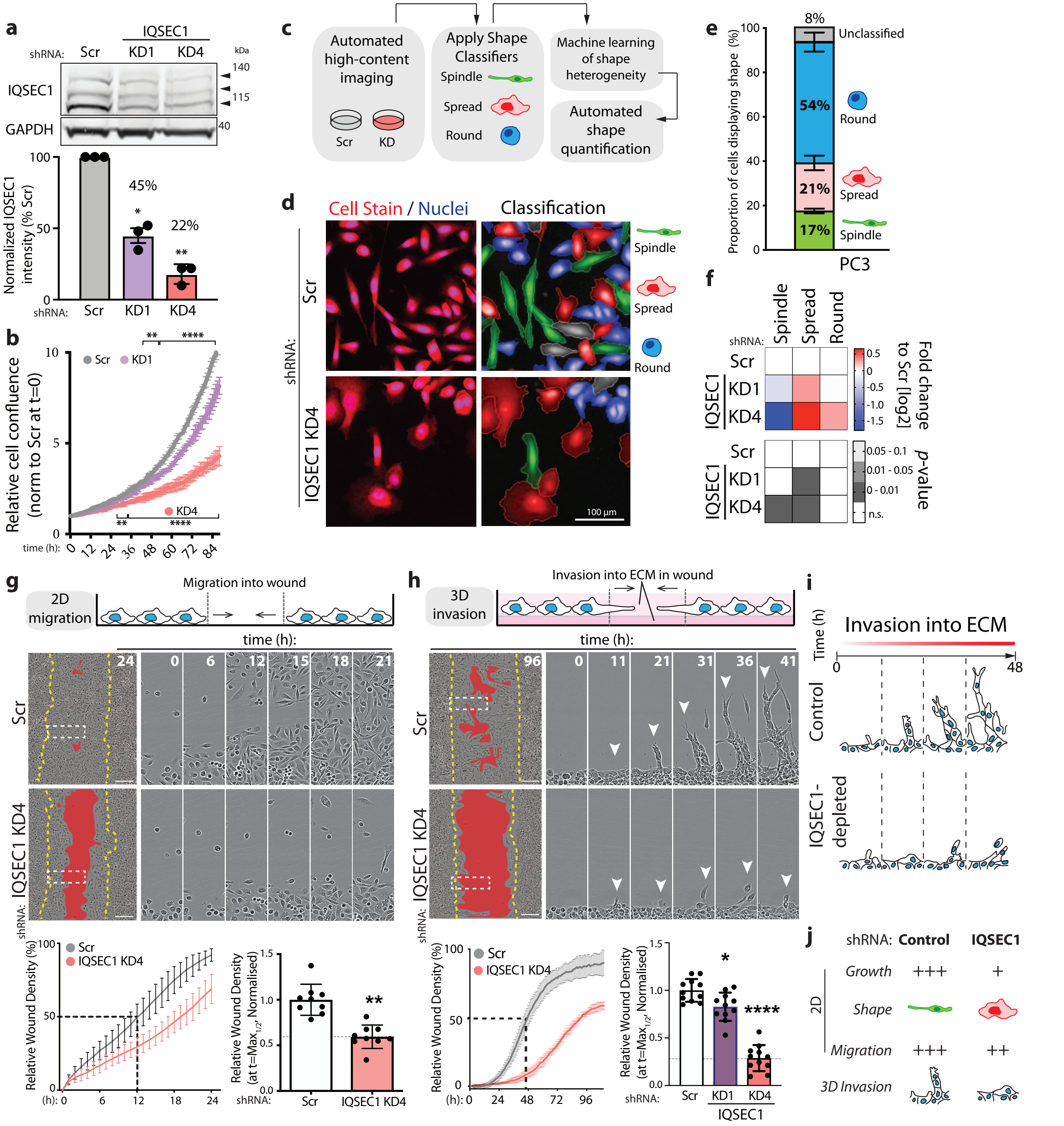

Supplementary Figure 1

Supplementary Figure 1. IQSEC1 is a regulator of cell elongation and collective invasion

(a) Western blot of PC3 cells expressing Scrambled (Scr) or *IQSEC1* (KD1, KD4) shRNAs using anti-IQSEC1 and GAPDH (loading control for IQSEC1) antibodies. Arrowheads, reduction of three major bands. IQSEC1 intensity for all bands combined normalised to Scr is shown. Mean ± s.d., n=3 independent experiments. p-values; Students t-test (2-tailed). *p≤0.05, **p≤0.01.

(b) PC3 cells expressing Scr or *IQSEC1* KD shRNA were imaged every hour. Cell confluence was measured and normalised to control. Mean ± s.d., n=3 biological experiment with 3 replicates/condition. p-values; Students t-test (2-tailed). **p≤0.01 and ***p≤0.001.

(c) Schematic of phenotypic analysis of PC3 cells in 2D. Machine learning applied to classify and quantify cells into three categories based on shape.

(d) PC3 cells expressing Scr or *IQSEC1* KD4 shRNA were stained with whole cell stain (WCS) (red) and Hoechst/nuclei (blue). Cells were classified into spindle, spread and round (green, red and blue respectively). n=4 independent experiments. Scale bar, 100μm.

(e-f) Proportion of PC3 cells with each phenotype is shown as (e) mean ± s.d. or (f) classified into round, spindle or spread. Heatmap indicates log2 fold change over Scr (upper heatmap). p-values (one-way ANOVA): greyscale values as indicated (lower heatmap). n=4 independent experiments, 10 replicates per condition, minimum 118,000 cells imaged/condition in total.

(g-h) Schema, PC3 cells plated for 24 hours before the resultant monolayers were (g) wounded or (h) wounded and overlaid with 25% ECM for 1 hour prior to imaging. Phase contrast images are shown where yellow lines indicate initial scratch wound and red pseudo colour shows wound after 24 hours. Scale bars, 300μm. Magnified images of boxed areas are shown. Relative wound density (RWD) was calculated at each time point (lower left panels). RWD at the time point where the Scr controls are 50% closed (t=$Max_{1/2}$) is shown (lower right panels). Samples were normalised to the average of all Scr controls across experiments. Mean ± s.d., n=3 independent experiments, 3 replicates/experiment in (g) and 4 in (h). p-values; Student's t-test (2-tailed). *p≤0.05, **p≤0.01 and ****p≤0.0001.

(i) Schema, depicts invasive ability of PC3 in ECM +/- IQSEC1.

(j) Schema, summarizes the effect of IQSEC1 depletion.

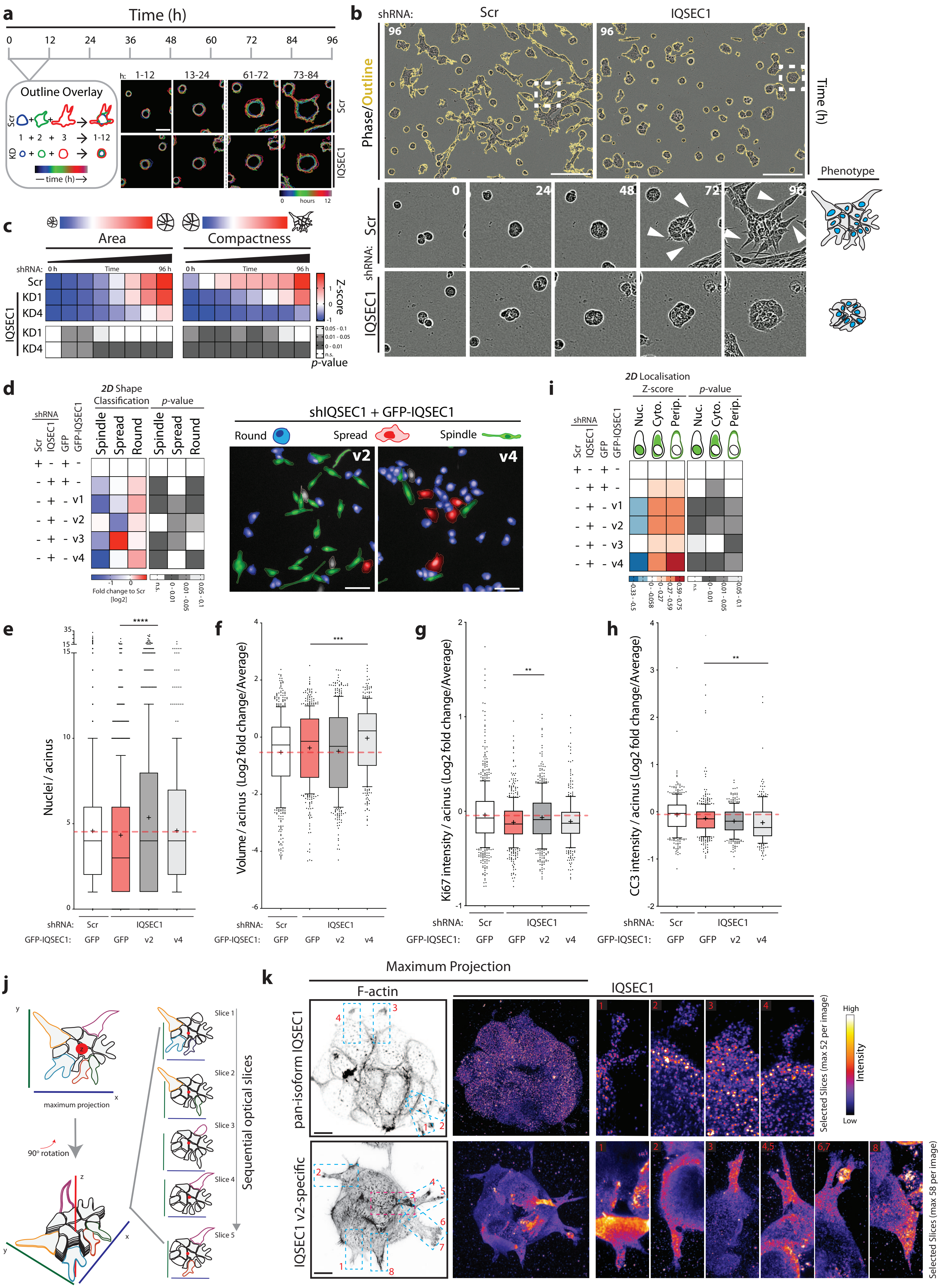

Supplementary Figure 2

Supplementary Figure 2. IQSEC1 v2 induces spindle shape and protrusive invasion

(a) Schema, outlines were colour-coded at each time point and overlaid in 12 hour blocks. Scale bars, 20µm.

(b) Phase contrast images are shown for PC3 acini expressing Scr or *IQSEC1* KD4 shRNA. Acini outlines, yellow and white arrowheads, protrusions. Magnified images are shown. Scale bars, 100µm.

(**c**) Quantitation of images in (b). CellProfiler was used to measure invasion parameters, over 4 days, in 12 hour blocks. Heatmap, area and compactness measurements Z-score-normalised to control. p-values; one-way ANOVA, greyscale values as indicated. n=4 independent experiments, 4 replicates/condition, 1,555-2,035 acini/condition.

(d) 2D PC3 cells expressing GFP or GFP-IQSEC1 v1-4 and either Scr or *IQSEC1* KD4 shRNA classified into spindle, spread and round phenotypes (green, red and blue respectively). Scale bars, 100µm. Quantitation is shown in heatmaps as log2-normalised fold change of each phenotype over control (top row in left heatmap). p-values; one-way ANOVA, greyscale values as indicated. n=2 independent experiments, 4 replicates/condition, 1,431-5,587 cells quantified/condition in total.

(**e-h**) PC3 acini expressing GFP or GFP-IQSEC1 v2 or v4 and either Scr or *IQSEC1* KD4 shRNA were analysed for (e) Nuclei number, (f) Acinus volume (μm^3), (g) proliferation (Ki67) or (h) apoptosis (CC3, Cleaved Caspase 3). In (f-h) values, log2-normalised fold change to average. Box-and-whiskers plots: 10–90 percentile; +, mean; dots, outliers; midline, median; boundaries, quartiles. n=2 independent experiments with 4 replicates/condition. (e) 462-1,669, (f) 335-610, (g) 365-724 and **(h)** 246-463 cells quantified/condition in total. p-values; one-way ANOVA. $^{**}p \leq 0.01$, $^{***}p \leq 0.001$ and $^{****}p \leq 0.0001$.

(i) 2D PC3 cells expressing GFP or GFP-IQSEC1 v1-4 and either Scr or *IQSEC1* KD4 shRNA were analysed for nuclear, cytoplasmic and cortical localisation of GFP using Harmony High-Content Imaging and Analysis Software. Quantitation is shown in heatmaps. p-values; one-way ANOVA, greyscale values as indicated. n=2, 4 replicates/condition.

(j) Schema, depicts how optical slices were combined to form maximum projections of acini body and protrusions.

(k) Maximum projections of PC3 acini fixed and stained with F-actin (black) and an IQSEC1 antibody that detects all variants or one that is specific for v2. Localisation of IQSEC1 can be appreciated from FIRE LUT. n=3 independent experiments. Scale bars, 10μm.

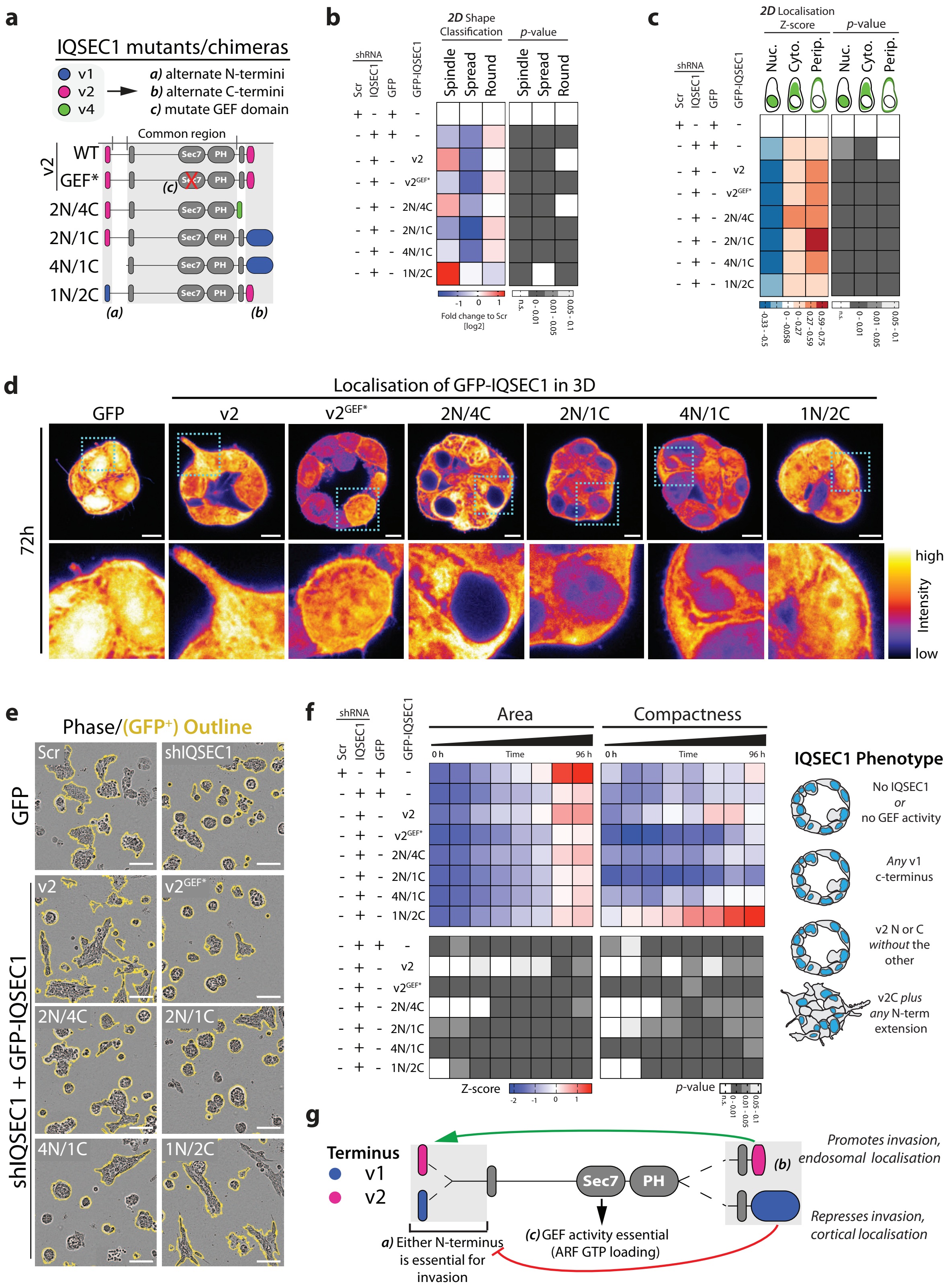

a
IQSEC1 mutants/chimeras
v1
v2
v4
a) alternate N-termini
b) alternate C-termini
c) mutate GEF domain
Common region
WT
GEF*
2N/4C
2N/1C
4N/1C
1N/2C
Sec7
PH
b
2D Shape Classification
p-value
shRNA
Scr
IQSEC1
GFP
GFP-IQSEC1
Spindle
Spread
Round
Fold change to Scr [log2]
c
2D Localisation Z-score
Nuc.
Cyto.
Perip.
d
Localisation of GFP-IQSEC1 in 3D
GFP
v2
2N/4C
2N/1C
4N/1C
1N/2C
72h
high
Intensity
low
e
Phase/(GFP+) Outline
Scr
shIQSEC1
shIQSEC1 + GFP-IQSEC1
f
Area
Compactness
Time
0 h
96 h
Z-score
p-value
IQSEC1 Phenotype
No IQSEC1 or no GEF activity
Any v1 c-terminus
v2 N or C without the other
v2C plus any N-term extension
g
Terminus
v1
v2
Promotes invasion, endosomal localisation
Represses invasion, cortical localisation
a) Either N-terminus is essential for invasion
(c) GEF activity essential (ARF GTP loading)

Supplementary Figure 3. N-terminal common extension in IQSEC1 v2 confers enhanced invasive activity in a GEF-dependent manner

(a) Schemes, IQSEC1 chimeras generated by swapping N- and C- terminal domains from IQSEC1 variants v1-v4. Colour coding as follows: v1 = blue, v2 = pink and v4 = green. Chimeras with alternate N-termini, C-termini or are GEF dead are labelled (a), (b) or (c) respectively.

(b) PC3 cells expressing GFP, GFP-IQSEC1 v2 or GFP-IQSEC1 chimeras and either Scr or *IQSEC1* KD4 shRNA were classified into spindle, spread and round phenotypes. Heatmap indicates log2 fold change over Scr (upper heatmap). p-values; one-way ANOVA, greyscale values as indicated (lower heatmap). n=2 independent experiments, 4 replicates/condition, 10,000-35,000 cells/condition in total.

(c) Cells from (b) were also analysed for nuclear, cytoplasmic and cortical localisation and quantitation is shown in heatmaps. p-values; one-way ANOVA, greyscale values as indicated (lower heatmap). n=2 independent experiments, 4 replicates/condition.

(d) PC3 acini expressing GFP, GFP-IQSEC1 v2 or GFP-IQSEC1 chimeras were fixed after 3 days and FIRE LUT used to show localisation and intensity of GFP. Magnified images of boxed regions are shown (lower panels). n=3 independent experiments. Scale bars, 10µm.

(e-f) Phase images from PC3 acini expressing GFP, GFP-IQSEC1 v2 or GFP-IQSEC1 chimeras and either Scr or *IQSEC1* KD4 shRNA are shown after 96 hours. GFP-positive acini are outlined in yellow. Scale bars, 100µm. Heatmap, area and compactness measurements Z-score-normalised to control. p-values; one-way ANOVA, greyscale values as indicated. n=2 independent experiments, 4 replicates/condition, 550-1,662 acini/condition in total. Cartoon, depicts acini phenotype representative of each condition.

(g) Schema, depicts the role of IQSEC1 v1 and v2 termini on localisation and invasion.

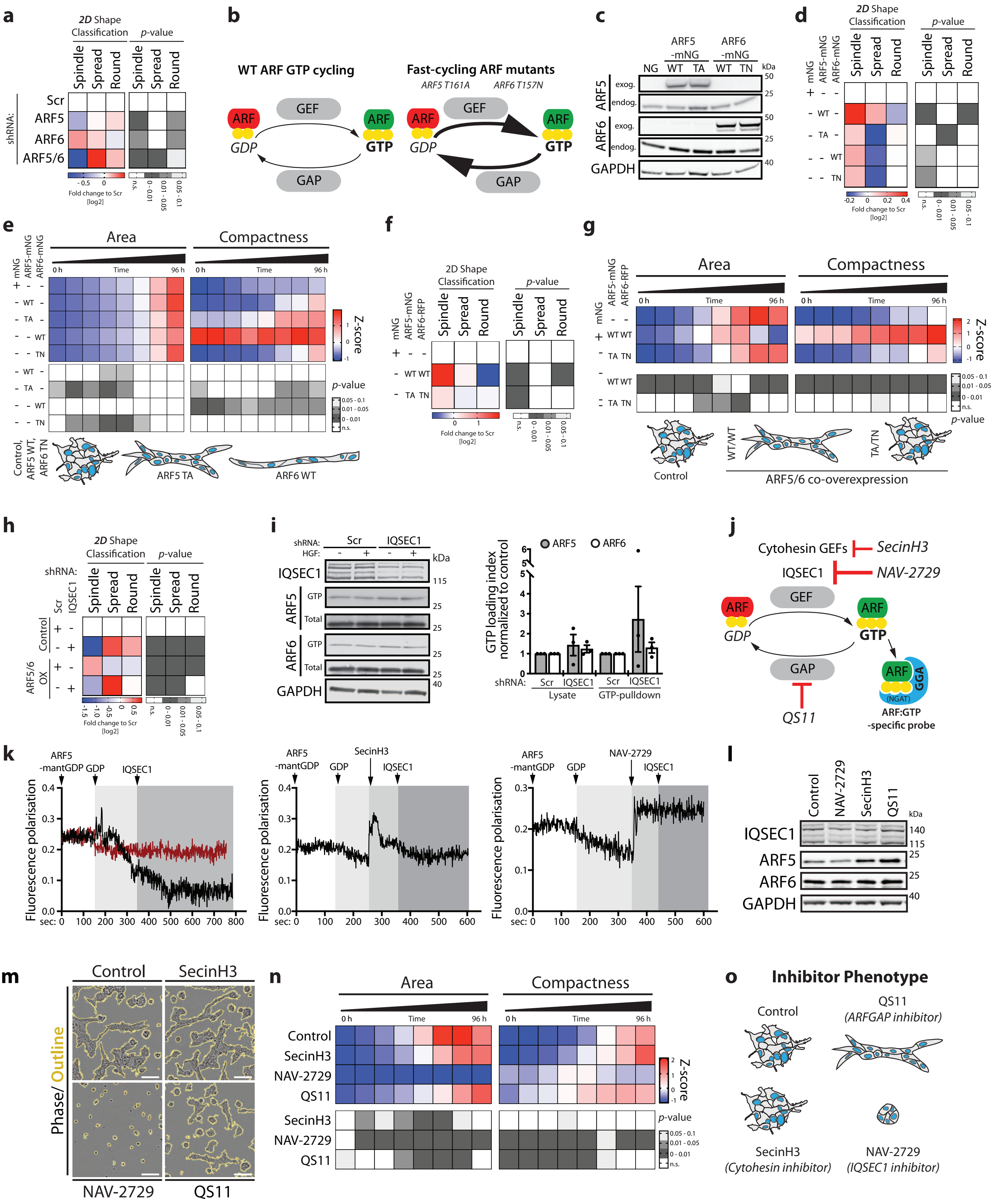

Supplementary Figure 4

Supplementary Figure 4. ARF5 and ARF6 co-operate to regulate both growth and invasion

(a) Cells expressing Scr, *ARF5, ARF6* or *AR5/6* shRNA. Heatmaps show log2 fold change over control. p-values; one-way ANOVA, greyscale values indicated. n=2 independent experiments, 4 replicates, minimum of 4,000 cells/condition analysed.

(b) Schema, comparison of ARF activation.

(c) Western blot of cells expressing mNG, ARF5-mNG, ARF5-mNG TA, ARF6-mNG or ARF6-mNG TN using anti-ARF5, ARF6 and GAPDH (loading control for ARF5) antibodies.

(d) Quantitation of cells (c). Heatmaps show log2-normalised fold change over control. p-values; one-way ANOVA, greyscale values indicated. n=2, 4 replicates.

(e) Quantitation of acini (c). Heatmap, area and compactness measurements Z-score-normalised to control. p-values; one-way ANOVA, greyscale values indicated. n=2 independent experiments with 4 replicates/condition. Minimum of 900 acini/condition analysed. Cartoon, representative acini.

(f) Quantitation of cells expressing mNG, ARF5-mNG and ARF6-RFP or ARF5-mNG TA and ARF6-RFP TN. Heatmaps show log2 fold change over control. p-values; one-way ANOVA, greyscale values indicated. n=3 independent experiments, 3 replicates.

(g) Quantitation of acini. Heatmaps show measurements as Z-score-normalised values. p-values (one-way ANOVA): greyscale values indicated. n=2 independent experiments, 4 replicates. Cartoon, representative acini.

(h) Quantitation of cells expressing control or ARF5/ARF6 and Scr or *IQSEC1* KD4 shRNA. Heatmap, area and compactness measurements Z-score-normalised to control. p-values; one-way ANOVA, greyscale values indicated. n=2 independent experiments, 4 replicates.

(i) Cells expressing Scr or *IQSEC1* shRNA were serum starved and HGF added. GGA3 pulldown and western blot performed using anti-IQSEC1, ARF5, ARF6, and GAPDH (loading control for ARF6) antibodies. Loading normalised to control is shown. Mean ± s.d., n=3 independent experiments. p-values; one-way ANOVA, n.s.

(j) Schema, modulation of ARF activity.

(k) Polarisation assay to study exchange of fluorescent nucleotides (MANT) on ARF5. IQSEC1 v2 SEC7-PH WT and IQSEC1 v2 SEC7-PH GEF* (GEF dead) used to test role of IQSEC1 in ARF5 activation. Changes in polarisation over time shown.

(l) Western blot of cells treated with inhibitors using anti-IQSEC1, ARF5, ARF6 and GAPDH (loading control for IQSEC1) antibodies. n=2 independent experiments

(m-n) Images of acini treated with inhibitors. Scale bars, 100µm. Heatmap, area and compactness measurements Z-score-normalised to control. p-values; one-way ANOVA, greyscale values indicated. n=1 experiment, 4 replicates.

(o) Cartoon, representative acini.

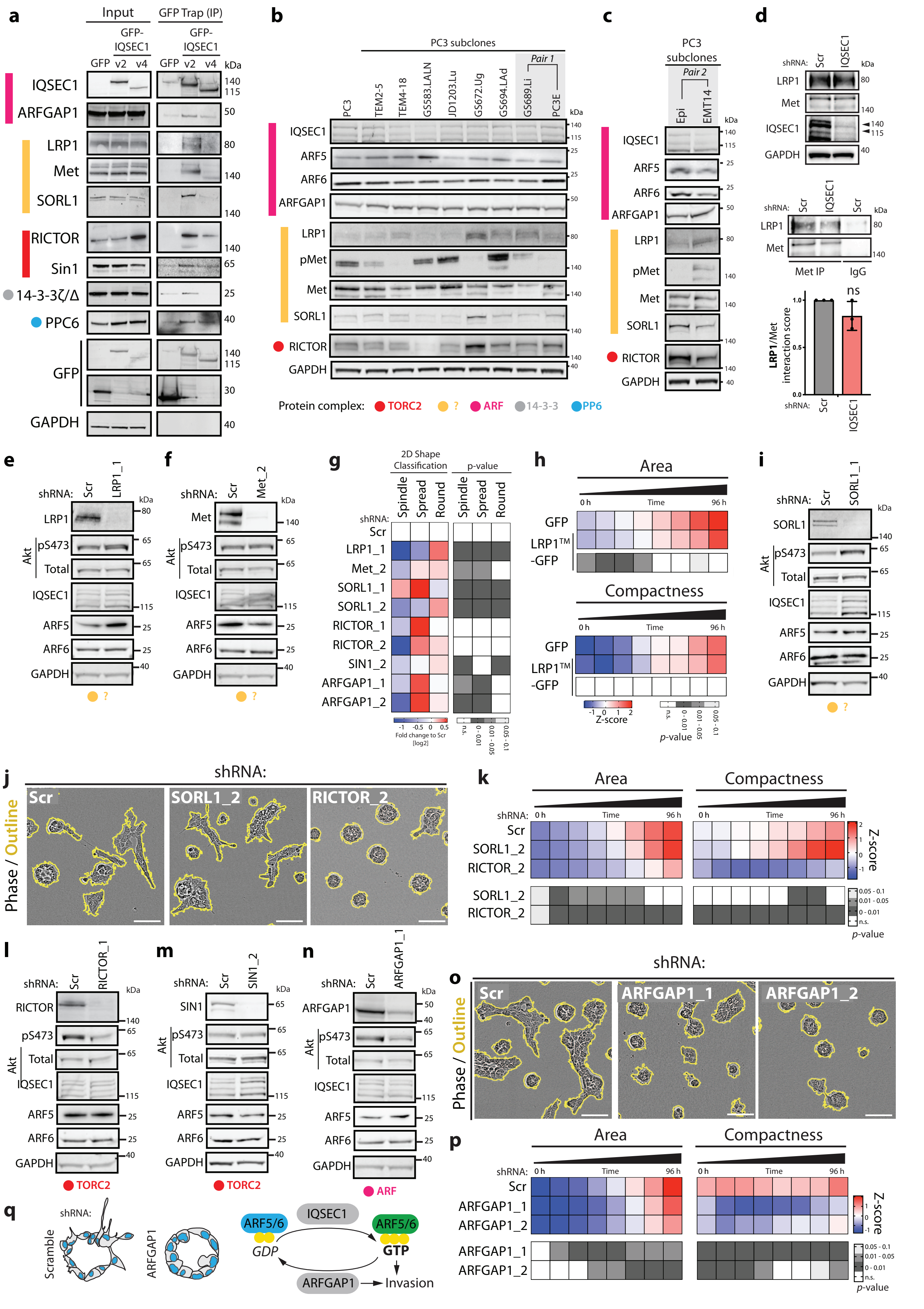

Supplementary Figure 5

Supplementary Figure 5. IQSEC1 interacts with multiple transmembrane proteins

(a) GFP-trap immunoprecipitation on cells expressing GFP, GFP-IQSEC1 v2 or v4. Western blot performed using anti-IQSEC1, LRP1, Met, SORL1, RICTOR, Sin1, ARFGAP1, 14-3-3ζ/Δ, PPC6, GFP and GAPDH (loading control for GFP) antibodies. n=3 independent experiments.

(b) Western blot of PC3 subclones using anti-IQSEC1, ARF5, ARF6, ARFGAP1, LRP1, phospho-Y1234/1235 Met, Met, SORL1, RICTOR and GAPDH (loading control for IQSEC1) antibodies. n=2 independent experiments.

(c) Western blot of Epi and EMT14 using anti-IQSEC1, ARF5, ARF6, ARFGAP1, LRP1, phospho-Y1234/1235 Met, Met, SORL1, RICTOR and GAPDH (loading control for IQSEC1) antibodies. n=2 independent experiments.

(d) Met immunoprecipitation on cells expressing Scr or *IQSEC1* KD4 shRNA. Western blotting performed using anti-LRP1, Met, IQSEC1 and GAPDH (loading control for IQSEC1) antibodies. Quantitation of LRP1/Met interaction normalised to Scr shown. Mean ± s.d., n=3 independent experiments. p values: one-way ANOVA. n.s.

(e-f) Western blot of PC3 cells expressing Scr and (e) *LRP1* and (f) *Met* shRNA. GAPDH is the loading control for Akt. n=2 independent experiments.

(g) Quantitation of cells expressing shRNAs for IQSEC1 binding partners as log2 fold change of each phenotype over control. p-values; one-way ANOVA, greyscale values indicated. n=2, 4 replicates per condition, minimum 14,000 cells imaged/condition.

(h) Quantitation of acini expressing GFP or LRP1-GFP. Heatmap, area and compactness measurements Z-score-normalised to control. p-values; one-way ANOVA, greyscale values indicated. n=3 independent experiments, 3 replicates, minimum 150 acini/condition.

(i) Western blot of cells expressing *SORL1* shRNA. GAPDH is the loading control for Akt. n=2 independent experiments.

(j-k) Images of acini expressing Scr (also in 4h), *SORL1*_2 and *RICTOR_2* shRNA. Scale bars, 100µm. Heatmap, area and compactness measurements Z-score-normalised to control. p-values; one-way ANOVA, greyscale values

indicated. n=2 independent experiments, 4 replicates, minimum 700 acini/condition.

(l-n) Western blots of cells expressing (l) *RICTOR* or (m) *SIN1* or (n) *ARFGAP1* shRNA. GAPDH is the loading control for Akt. n=2 independent experiments.

(o-p) Images of acini expressing Scr and *ARFGAP1* shRNA. Scale bars, 100µm. Heatmap, area and compactness measurements Z-score-normalised to control. p-values; one-way ANOVA, greyscale values indicated. n=2 independent experiments, 4 replicates, minimum 1,100 acini/condition.

(q) Cartoon, depicts representative acini upon depletion of ARFGAP1.

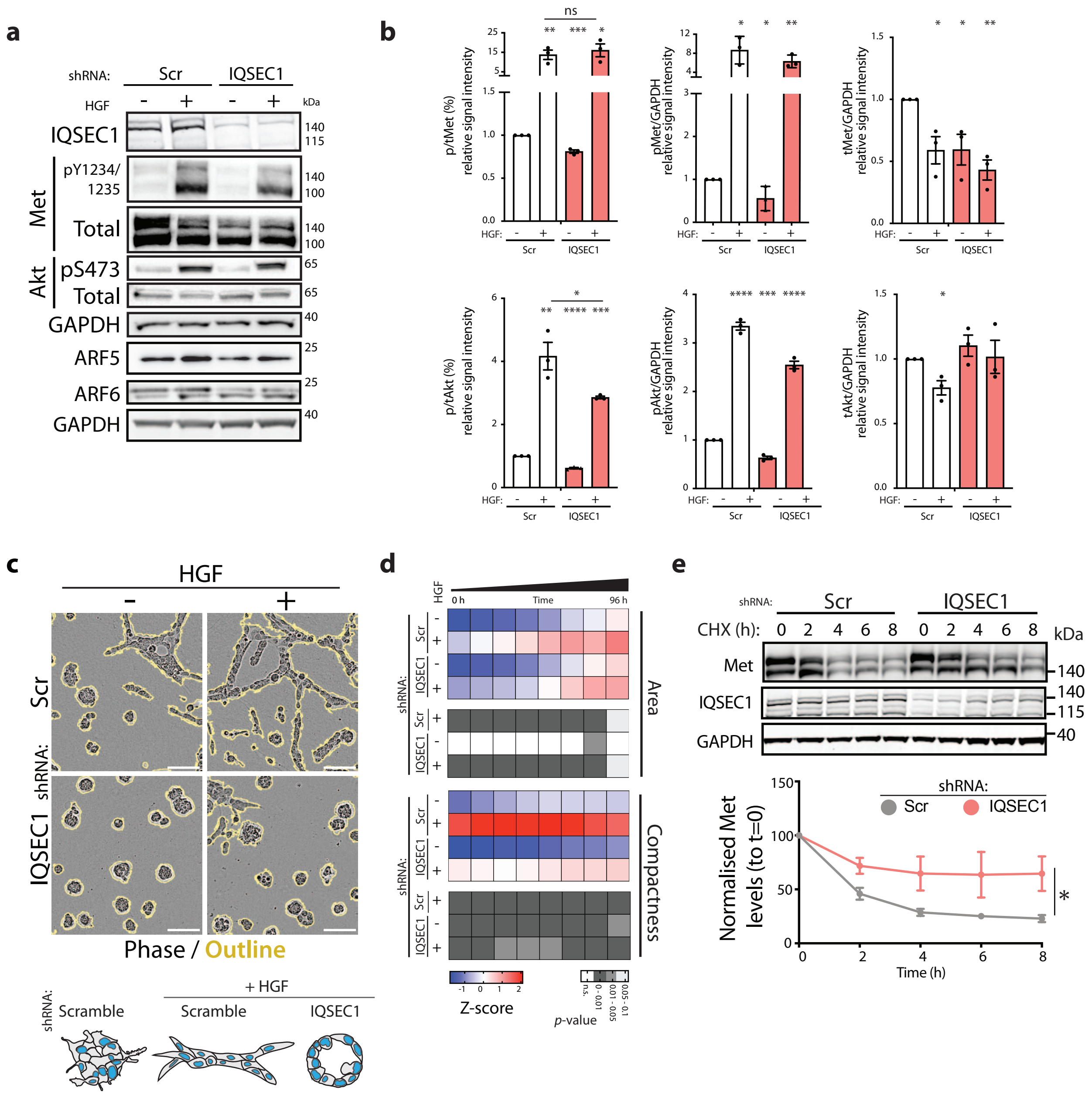

Supplementary Figure 6

Supplementary Figure 6. HGF stimulation is abrogated by IQSEC1 depletion

(a) Western blot of PC3 cells expressing Scr or *IQSEC1* KD4 shRNA stimulated with HGF for 30 minutes using anti-IQSEC1, phospho-Y1234/1235 Met, Met, phospho-S473 Akt, Akt, ARF5, ARF6 and GAPDH (loading control for Akt blot and sample control for ARFs) antibodies.

(b) Quantitation of Met and Akt: phospho/total, phospho/GAPDH and total/GAPDH. Expression is presented as signal intensity relative to control. Mean ± s.d., n=3 independent experiments. p-values; Student's t-test (2-tailed). *p≤0.05, **p≤0.01, ***p≤0.001 and ****p≤0.0001.

(c-d) Phase contrast images of PC3 acini expressing Scr or *IQSEC1* KD4 shRNA stimulated with HGF for 96 hours. Scale bars, 100μm. Cartoon, depicts acini phenotype representative of each condition. Heatmap, area and compactness measurements Z-score-normalised to control. p-values; one-way ANOVA, greyscale values as indicated. n=3 independent experiments, 4 replicates/condition, 1,400 - 4,025 acini/condition in total.

(e) Western blots of PC3 cells expressing Scr or *IQSEC1* KD4 shRNA treated with cycloheximide (CHX) for various times using anti-IQSEC1, Met and GAPDH (shown for Met blot) antibodies. Quantitation of Met expression levels normalised to time 0 is shown. Mean ± s.e., n=3 independent experiments. p-values; one-way ANOVA. *p≤0.05, ***p≤0.001 and ****p≤0.0001.

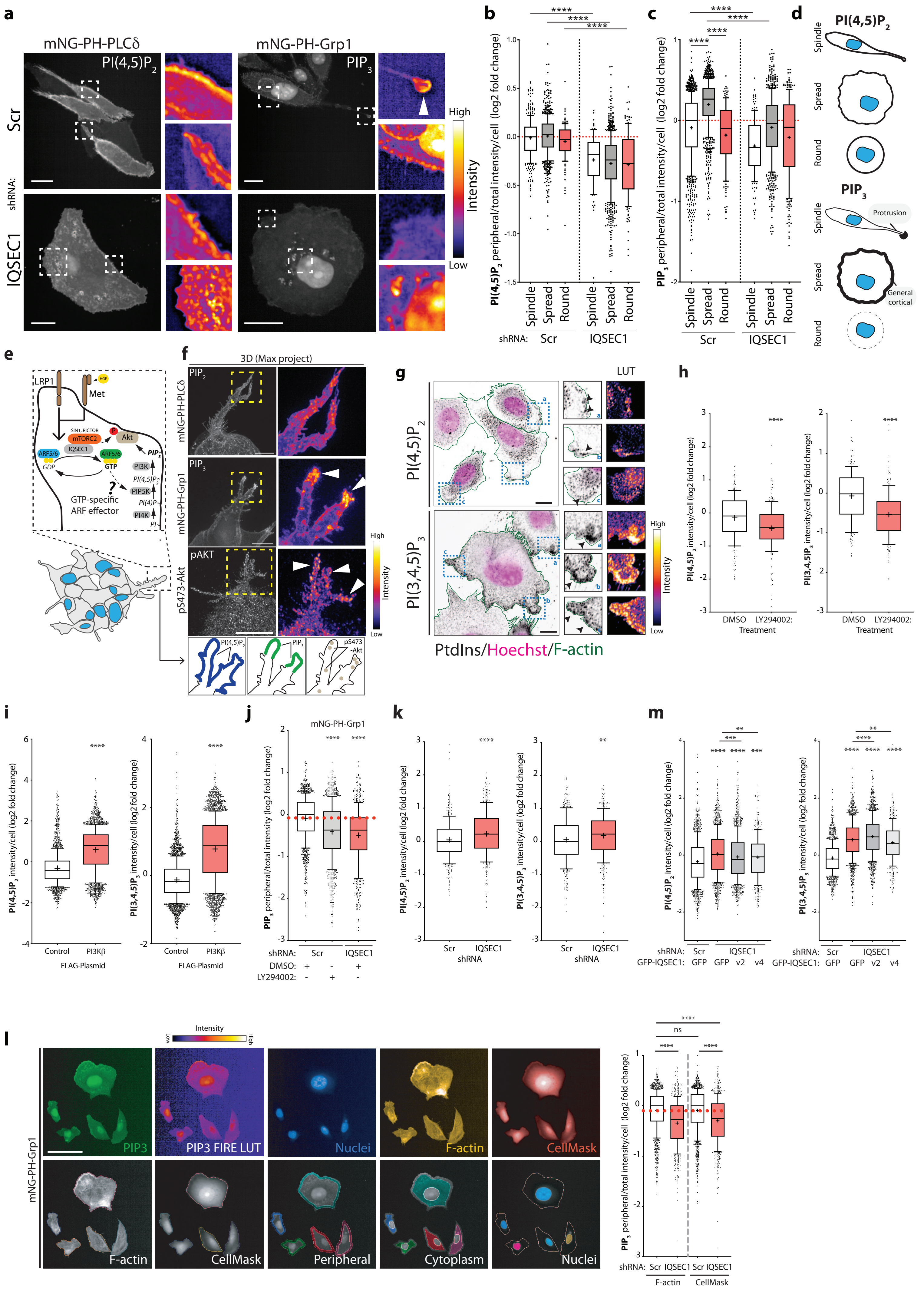

Supplementary Figure 7

Supplementary Figure 7. IQSEC1-ARF controls cortical PI(4,5)P_2 generation, which is required for production of PIP_3

(a-c) Cells expressing mNG PH-PLCδ (PIP_2) or PH-Grp1 (PIP_3) and Scr or *IQSEC1* shRNA KD4 were fixed. FIRE LUT, localisation of GFP. Arrowhead, peripheral localisation. n=3 independent experiments. Scale bars, 20μm. (b-c) Quantitation shown. n=3 independent experiments, 4 replicates/condition/experiment. 1,330/1,049 and 2,222/1,182 cells analysed for Src/*IQSEC1* KD4 shRNA. p-values; one-way ANOVA.

(d) Cartoon, summary of PIP localization.

(e) Schema, signalling pathways in acini.

(f) Acini described in (a) stained with phospho-S473 Akt antibody. FIRE LUT, localisation of mNeonGreen/pAkt. Magnified images shown. Arrowheads, localisation. Scale bars, 10 μm. Cartoon, spatial PIP production.

(g) Cells stained with anti-PI(4,5)$P_{2,}$ PI(3,4,5)P_3 antibodies, F-actin (green outlines) and Hoechst (magenta). Magnified images and FIRE LUT shown. Arrowheads, peripheral localization. Scale bars, 10μm.

(h-i) Quantitation of total intensity/cell of anti-PI(4,5)$P_{2,}$ or PI(3,4,5)P_3 antibodies in cells treated with (h) LY294002 or (i) expressing PI3K-FLAG. (h) n=1, 4 replicates/condition, 221/246 (-/+ LY294002) cells/condition for PI(4,5)P_2 and 246/223 (-/+ LY294002) cells/condition for PI(3,4,5)$P_{3.}$ (i) n=3, 4 replicates/condition, 2,391/3,082 (-/+ PI3K-FLAG) cells/condition for PI(4,5)P_2 and 3,826/3,453 (-/+ PI3K-FLAG) cells/condition for PI(3,4,5)P_3. p-values; Students t-test (2-tailed).

(j) Cells expressing mNG PH-Grp1 (PIP_3) -/+ *IQSEC1* were treated with LY294002 and stained with F-actin, CellMask and Hoechst. Quantitation of peripheral/total PIP_3 intensity/cell shown. n=2 independent experiments, 4 replicates/condition, 1,410, 1,224 and 684 cells/condition. p-values; one-way ANOVA.

(k) Quantitation of total intensity/cell of anti-PI(4,5)P_2 or PI(3,4,5)P_3 antibodies -/+ *IQSEC1* shown. n=3 independent experiments, 3 replicates/condition, 466, 499, 470 and 478 cells/condition quantified. p-value; Students t-test (2-tailed).

(l) Cells expressing mNG PH-Grp1 (PIP_3) -/+ *IQSEC1* stained with F-actin, CellMask and Hoechst. Scale bar, 100μm. Quantitation of peripheral/total

PIP_3 intensity/cell shown. n=1 experiment, 4 replicates/condition, 1,572, 720, 1,567, 722 cells/condition in total. p-values; one-way ANOVA.

(m) Quantitation of total intensity/cell of anti-PI(4,5)P_2 or PI(3,4,5)P_3 antibodies in cells expressing GFP, GFP-IQSEC1 v2 or GFP-IQSEC1 v4 and Scr or *IQSEC1* KD4 shRNA. n=2 independent experiments, 4 replicates/condition, 1,630, 1,572, 1,539 and 511 (PI(4,5)P_2) and 1,677, 1,578, 1,549 and 534 (PI(3,4,5)P_3) GFP + cells/condition quantified. p-values; one-way ANOVA.

Box-and-whiskers plots: 10–90 percentile; +, mean; dots, outliers; midline, median; boundaries, quartiles. p-values: n.s. not significant, *p≤0.05, **p≤0.01, ***p≤0.001 and ****p≤0.0001.

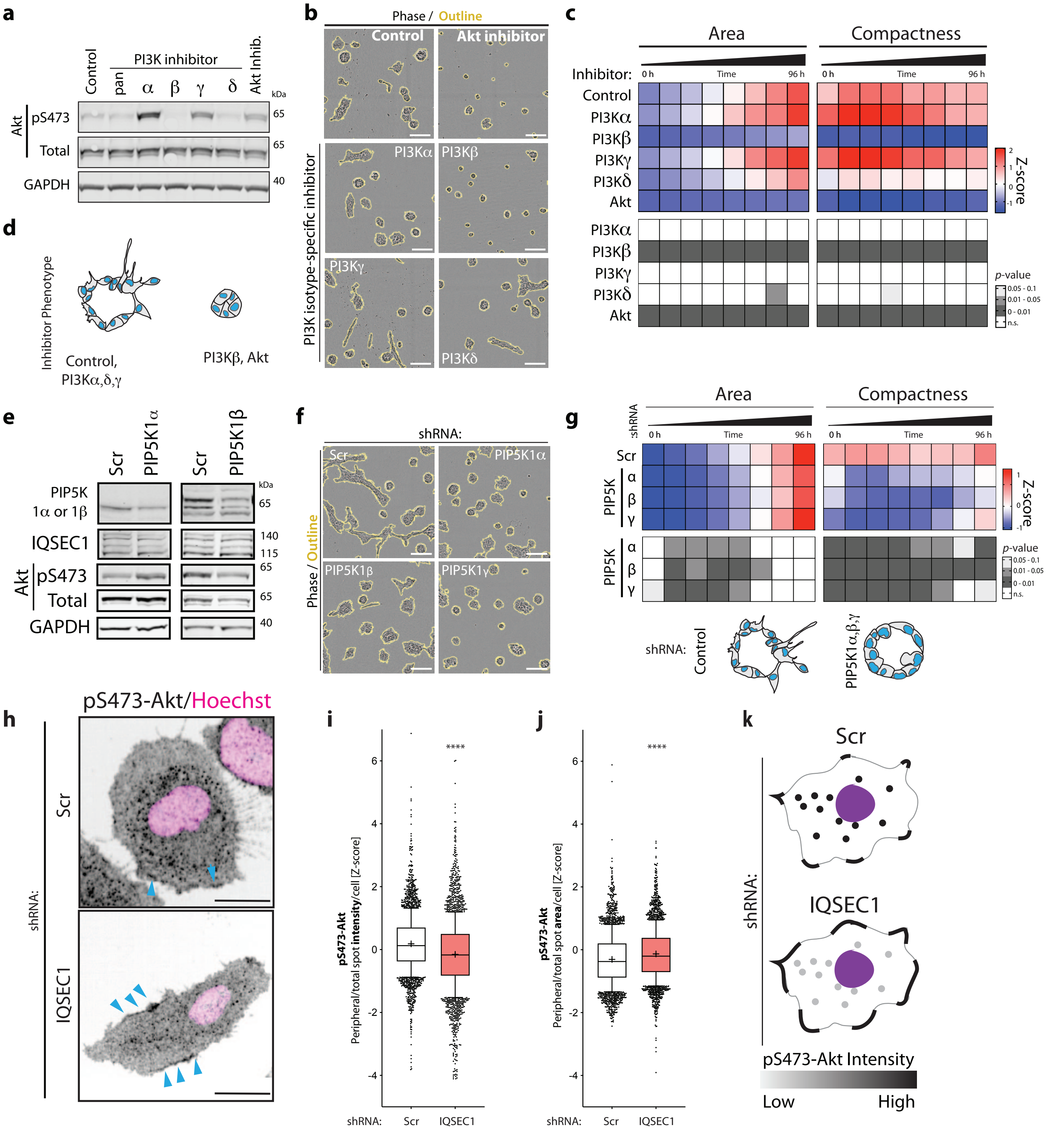

Supplementary Figure 8

Supplementary Figure 8. PIP5K1β-PI3Kβ-Akt pathway involved in 3D growth and invasion

(a) Western blot of PC3 cells treated with LY294002 (pan PI3K), AZD8835 (PI3Kα), AZD8186 (PI3Kβ), AS605240 (PI3Kγ), Cal-101 (PI3Kδ) and AktII (Akt) inhibitors for 24 hours. Anti-phospho-S473 Akt, Akt and GAPDH antibodies were used. n=2 independent experiments.

(b-c) Phase contrast images of PC3 acini described in **(a)**. Scale bars, 100μm. **(b)** Heatmaps show area and compactness measurements as Z-score-normalised values (upper heatmaps). p-values (one-way ANOVA): greyscale values as indicated (lower heatmaps). n=2 independent experiments, 4 replicates, minimum of 350 acini/condition in total. n=2 independent experiments.

(d) Cartoon, depicts acini phenotype representative of each condition.

(e) Western blot of PC3 cells expressing Scr, PIP5K1α or PIP5K1β shRNA using anti-PIP5K, IQSEC1, phospho-S473 Akt, Akt and GAPDH (loading control for Akt blots) antibodies. n=2 independent experiments.

(f-g) Phase contrast images of PC3 acini expressing Scr, PIP5K1α or PIP5K1β shRNA at 96 hours. Scale bars, 100μm. Heatmaps show area and compactness measurements as Z-score-normalised values to control. p-values (one-way ANOVA): greyscale values as indicated (lower heatmaps). n=2 independent experiments, 4 replicates, minimum of 1,348 acini/condition in total. Cartoon, depicts acini phenotype representative of each condition.

(h) PC3 cells expressing Scr or *IQSEC1* KD4 shRNA were stained for pS473 Akt (black) and Hoechst /nuclei (magenta). Blue arrowheads indicate localisation of active Akt. Scale bars, 10μm.

(i-j) Quantitation of pAkt (i) mean cortical/total intensity/cell or (j) mean cortical/total spot area/cell of PC3 cells expressing either Scr or IQSEC1 KD4 shRNA. Box-and-whiskers plots: 10–90 percentile; +, mean; dots, outliers; midline, median; boundaries, quartiles. n=3 independent experiments, 4 replicates/condition, 2,765 (Scr) and 2,837 (*IQSEC1*) cells/condition in total. p values; one-way ANOVA). ****$p \leq 0.0001$.

(k) Cartoon, depicting phospho-Akt intensity, in the presence or absence of IQSEC1, at different subcellular locations.

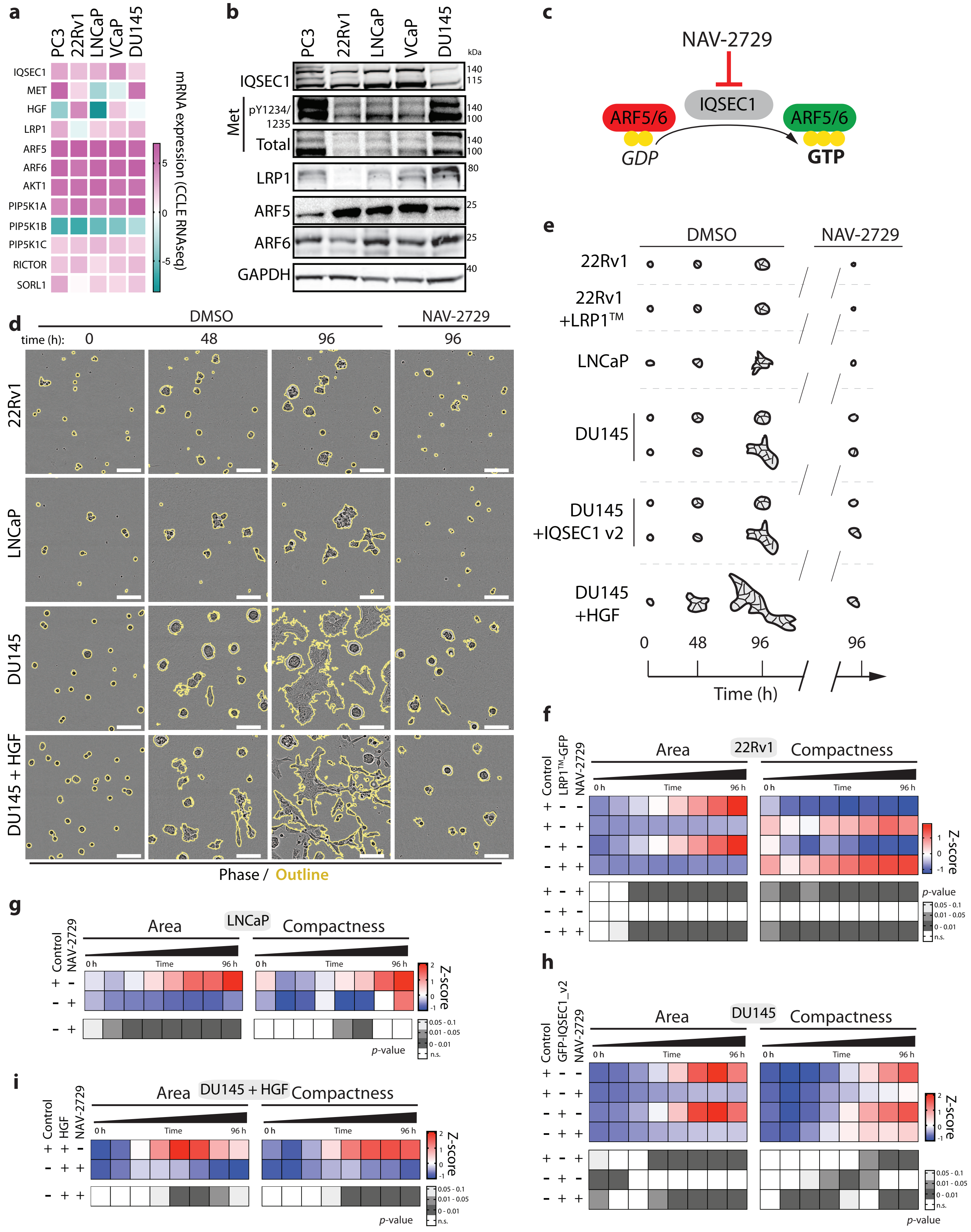

Supplementary Figure 9

Supplementary Figure 9. IQSEC1 regulates growth and invasion in multiple prostate cancer cell lines

(a) Heatmap shows mRNA expression levels, mined from CCLE, of the Met-PI3K-Akt pathway across different prostate cancer cell lines.

(b) Western blot of prostate cancer cell lines using anti-IQSEC1, phospho-Y1234/1234 Met, total Met, LRP1, ARF5, ARF6 and GAPDH antibodies. GAPDH is a loading control for ARF6 blot. n=2 independent experiments.

(c) Schema, effect of NAV-2729 on ARF GTPase cycle.

(d) Phase images of acini (- and + HGF) at different time points are shown. Acini were also treated with NAV-2729 for 96 hours. n=2 independent experiments. Scale bars, 100µm.

(e) Schema, summarizes the effect of each treatment described in (d) on acini growth and invasion.

(f-i) Quantitation of (f) 22Rv1, (g) LNCaP, (h) DU145 and (i) DU145 + HGF acini formation in the absence or presence of NAV-2729. 22Rv1 and DU145 acini were also expressing LRP1-GFP and IQSEC1-GFP v2 respectively. Heatmap, area and compactness measurements Z-score-normalised to control. p-values; one-way ANOVA, greyscale values indicated. n=2 independent experiments, 3 replicates/condition, 400 - 1,600 acini/condition in total.

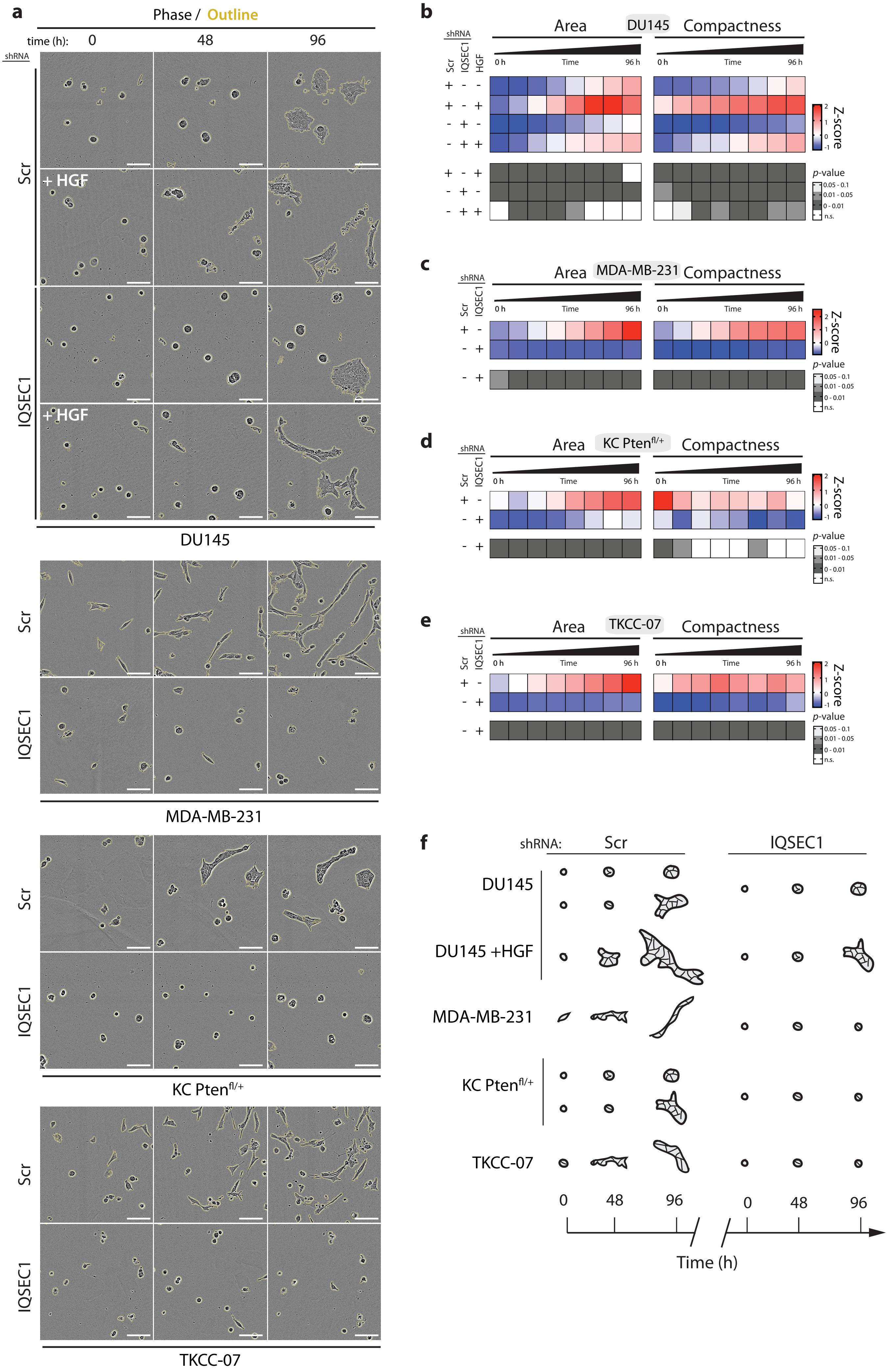

Supplementary Figure 10

Supplementary Figure 10. IQSEC1 regulates growth and invasion in multiple murine and human cancer cell lines

(a) Phase images of acini expressing either Scr or *IQSEC1* KD4 shRNA in DU145 (- and + HGF), MDA-MB-231, KC $Pten^{fl/+}$ or TKCC-07 are shown. Scale bars, 100µm.

(b-e) Quantitation of (b) DU145 (- and + HGF), (c) MDA-MB-231, (d) KC $Pten^{fl/+}$ and (e) TKCC-07 acini formation in the absence or presence of *IQSEC1* KD4 shRNA. Heatmap, area and compactness measurements Z-score-normalised to control. p-values; one-way ANOVA, greyscale values indicated. n=3 independent experiments, 4 replicates/condition, (b) 5,883 - 15,437, (c) 4,937 - 8,114, (d) 5,003 – 7,450 and (e) 2,608 – 5,211 acini/condition in total.

(f) Schema, summarizes the effect of IQSEC1 loss on acini growth and invasion.

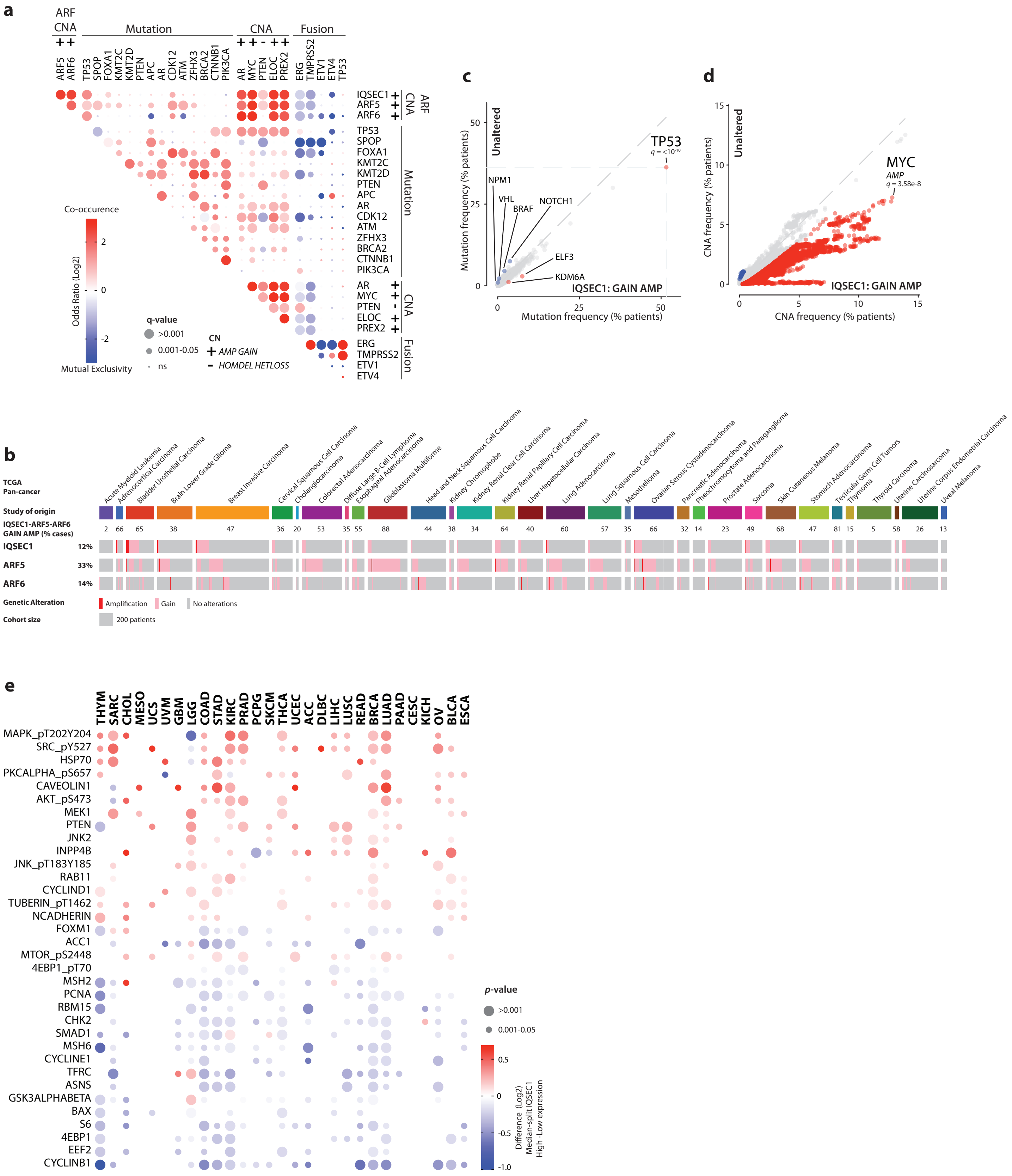

Supplementary Figure 11

Supplementary Figure 11. Additional characterisation of IQSEC1 association with clinical metrics

(a) Co-occurrence bubble plot matrix for IQSEC1, ARF5 and ARF6 CN gain compared to the 14 most common mutations, 6 CNAs, and five gene fusion events in prostate cancer. Colouring represents Log2 of odds ratio. Red, co-occurrence; blue, mutual exclusivity. Circle size, q-value. +, CN amplification (AMP) or GAIN; -, homozygous deletion (HOMDEL) or heterozygous Loss (HETLOSS). Q-values, one-sided Fisher exact test with Benjamini-Hochberg adjustment.

(b) CN increase frequencies (percentage of patients/cohort) in IQSEC1, ARF5, ARF6 across cancer types from pan-cancer TCGA analyses.

(c-d) Differential Copy Number Alteration (c) or (d) mutation frequencies between IQSEC1 CN-amplified patients compared to non-amplified comparison group. Red, IQSEC1 CN increase-associated. Blue, control group-associated. n=9,892 patients, across 32 tumour types. Q-values, one-sided Fisher exact test with Benjamini-Hochberg adjustment.

(e) Heatmap of protein changes related to IQSEC1 expression in patients. Values represent significant changes in protein/phospho-proteins profiled by RPPA analysis that are consistently altered in the same direction in at least a quarter of pan-cancer TCGA cohorts when patients were grouped by median split of IQSEC1 mRNA expression. Values are log2-transformed difference between a median split of total IQSEC1 mRNA levels. Red, co-occurring with high IQSEC1; blue, associated with low IQSEC1 levels. In RPPA, 190 protein/phospho-proteins profiled. Circle size, p-value. p-values, Independent Groups t-test. Patient sample n=7,790; See Table S4 for breakdown by tumour type.

Our nomenclature	UCSC Genome Browser	NCBI Nucleotide RefSeq	NCBI Nucleotide ID	NCBI Protein ID	Alternate IDs in literature	Additional IDs	Literature References
IQSEC1 V1	uc011auw.2	NM_001134382.3	IQSEC1a (Variant 1)	NP_001127854.1	BRAG2c		
IQSEC1 V2	uc003bxt.3	NM_014869.8	IQSEC1b (Variant 2)	NP_055684.3	BRAG2b		PMID: 16461286
IQSEC1 V3		AB018306.1		BAA34483.2	BRAG2a/GEP100	KIAA0763	PMID: 16461286
IQSEC1 V4	uc003bxu.4	NM_001330619.3	IQSEC1c (Variant 3)	NP_001317548.1	BRAG2d		

Supplementary Table 1

Supplementary Table 1. IQSEC1 variant nomenclature

Reference identification numbers and nomenclature for IQSEC1 variants.

row ID	isoform_uc003bxt_IsoformRatio_ControlDivided_Log2	isoform_uc003bxu_IsoformRatio_ControlDivided_Log2	isoform_uc011auw_IsoformRatio_ControlDivided_Log2	V2-V1 ratio_ControlDivided_Log2	gene_IQSEC1_ControlDivided_Log2	Unique count(PatientID)_NormalSamples	Unique count(PatientID)_TumourSamples
BLCA	-0.0454564	0.20961127	-0.903578	0.01902134	-0.6071592	19	408
BRCA	0.12263143	0.03454449	-0.6608392	0.19365703	-0.7028442	112	1093
CESC	-0.5212677	0.48714734	-0.8248277	-0.4466158	-1.3141784	3	304
CHOL	-0.4131846	0.14675817	0.38828484	-0.4314829	-0.8614579	9	36
COAD	0.39954908	0.00287994	-0.6058477	0.48181953	-0.1230543	41	285
ESCA	-0.3501124	0.15456968	-0.2538123	-0.3479711	-0.8978865	11	184
KICH	-0.0850435	0.15812894	-0.46684	-0.0147107	0.73155292	25	66
KIRC	0.02916776	-0.0689371	-0.1736231	0.04427441	-0.154391	72	533
LIHC	-0.1386565	-0.1777422	0.17569234	-0.1801919	0.38987016	50	371
LUAD	-5.082E-05	0.0106179	-0.3575599	0.02806778	-0.9288379	59	515
LUSC	0.00993884	0.04660915	-0.3854053	0.05541145	-1.5362055	51	501
PAAD	0.09697399	-0.1992505	0.05334858	0.06505223	-0.7390118	4	178
PCPG	0.20948245	-1.0802905	0.96939746	-0.0131824	-0.8046123	3	179
PRAD	0.09365186	0.06069443	-0.9541551	0.18619528	0.15270889	52	497
READ	0.32848927	0.03183749	-0.7116353	0.42549455	-0.1090786	10	94
SARC	-0.6768325	0.09248933	0.65797953	-0.8682658	0.34352391	2	259
STAD	-0.0572662	0.00248675	-0.1916982	-0.0304931	-0.2506005	35	415
THCA	0.18063912	-0.1589311	-0.1886437	0.14238164	-0.1577543	59	501
THYM	0.83039789	-0.6551475	-0.8693035	0.93627452	-0.4037472	2	120
UCEC	0.12332599	0.16477124	-1.0833349	0.28465008	-1.0040966	24	177
**Total**						**643**	**6716**

Supplementary Table 2

Supplementary Table 2. Normal versus tumour IQSEC1 variant and expression values and patient same sizes

Values corresponding to Bubble Heatmap in Supplementary Figure 8, listed by tumour type.

Target Gene	shRNA target sequence (5' - 3')
shScr	CCGCAGGTATGCACGCGT
IQSEC1 KD1	GATCTATGAACGGATCCGTAA
IQSEC1 KD4	CCAGTACCAGATGAACAAGAA
IQSEC1 KD murine	CCAGTGTTACTGTTGGCAAATCTC
Arf 5_1	GATGCAGTGCTGCTGGTATTT
Arf 6_3	GCTCACATGGTTAACCTCTAA
LRP1_1	GATGCCTATCTGGACTATATT
LRP1_2	GATCCGTGTGAACCGCTTTAA
MET_2	GTGTGTTGTATGGTCAATAAC
SORL1_1	GCCCAGTTTGTCACAAGACAT
SORL1_2	CCTATGCCATTGCTGTCTTTA
RICTOR_1	ACTTGTGAAGAATCGTATCTTCTC
RICTOR_2	GCAGCCTTGAACTGTTTAA
SIN1_2	CTAAGCAATCACGACTATAAA
ARFGAP1_1	GTGCAGGATGAGAACAACGTT
ARFGAP1_2	GCCAGTTCACTACGCAGTAT
PIP5K1α	GCTTCCAGGATACTACATGAA
PIP5K1β	ACGACAGGCCTAC ACTCTATT
PIP5K1γ	CAACACGGTCTTTCGGAAGAA

Supplementary Table 3

Supplementary Table 3. List of shRNA target sequences

TumorType	Unique count(SampleID)
ACC	46
BLCA	344
BRCA	892
CESC	173
CHOL	30
CORE	498
DLBC	33
ESCA	126
GBM	244
HNSC	212
KICH	63
KIRC	478
KIRP	217
LGG	435
LIHC	184
LUAD	365
LUSC	328
MESO	63
OV	436
PAAD	123
PCPG	82
PRAD	352
SARC	226
SKCM	356
STAD	392
TGCT	122
THCA	380
THYM	90
UCEC	440
UCS	48
UVM	12
**Total**	7790

Supplementary Table 4

Supplementary Table 4. Patient sample count for TCGA Pan-cancer RPPA data per tumour type
