# Supplementary material for: An ARF GTPase module promoting invasion and metastasis through regulating phosphoinositide metabolism
Source: Nat Commun. 2021 Mar 12;12:1623. doi: 10.1038/s41467-021-21847-4 (PMC7955138; doi:10.1038/s41467-021-21847-4)
Supplement: Supplementary file 3 — Description of Additional Supplementary Files [file 41467_2021_21847_MOESM3_ESM.pdf]

Description of Additional Supplementary Files

Supplementary Movie 1: The progressive development of PC3 acini from cell expressing Scrambled shRNA.

Supplementary Movie 2: The progressive development of PC3 acini from cell expressing *IQSEC1* KD4 shRNA.
