# Supplementary material for: An ARF GTPase module promoting invasion and metastasis through regulating phosphoinositide metabolism
Source: Nat Commun. 2021 Mar 12;12:1623. doi: 10.1038/s41467-021-21847-4 (PMC7955138; doi:10.1038/s41467-021-21847-4)
Supplement: Supplementary file 6 — Reporting Summary [file 41467_2021_21847_MOESM6_ESM.pdf]

nature research

Corresponding author(s): David Bryant

Last updated by author(s): Feb 4, 2021

Reporting Summary

Nature Research wishes to improve the reproducibility of the work that we publish. This form provides structure for consistency and transparency in reporting. For further information on Nature Research policies, see Authors & Referees and the Editorial Policy Checklist .

Statistics

For all statistical analyses, confirm that the following items are present in the figure legend, table legend, main text, or Methods section.

n/a	Confirmed	
[ ]	[x]	The exact sample size (*n*) for each experimental group/condition, given as a discrete number and unit of measurement
[ ]	[x]	A statement on whether measurements were taken from distinct samples or whether the same sample was measured repeatedly
[ ]	[x]	The statistical test(s) used AND whether they are one- or two-sided<br>*Only common tests should be described solely by name; describe more complex techniques in the Methods section.*
[ ]	[x]	A description of all covariates tested
[ ]	[x]	A description of any assumptions or corrections, such as tests of normality and adjustment for multiple comparisons
[ ]	[x]	A full description of the statistical parameters including central tendency (e.g. means) or other basic estimates (e.g. regression coefficient) AND variation (e.g. standard deviation) or associated estimates of uncertainty (e.g. confidence intervals)
[ ]	[x]	For null hypothesis testing, the test statistic (e.g. *F*, *t*, *r*) with confidence intervals, effect sizes, degrees of freedom and *P* value noted<br>*Give P values as exact values whenever suitable.*
[x]	[ ]	For Bayesian analysis, information on the choice of priors and Markov chain Monte Carlo settings
[x]	[ ]	For hierarchical and complex designs, identification of the appropriate level for tests and full reporting of outcomes
[x]	[ ]	Estimates of effect sizes (e.g. Cohen's *d*, Pearson's *r*), indicating how they were calculated

Our web collection on statistics for biologists contains articles on many of the points above.

Software and code

Policy information about availability of computer code

Data collection

Data from real-time imaging of 3D acini and invasion and migration assays was collected using Incucyte ZOOM Live Cell Analysis System Software 2018A (Essen Biosciences).

For high throughput image collection of 2D cell phenotypes and 3D acini cultures, we utilized an Opera Phenix Z9501 high-content imaging system with the associated software package Harmony (PerkinElmer, Version 4.6).

Super-resolution and confocal images were collected using an Zeiss 880 Laser Scanning Microscope with Airyscan and processed using ZEN 3.2 software.

Images from western blots were collected using a ChemiDoc Imager (BioRad) using Image Lab 6.1 software or an Odyssey Imaging System (LI-COR Biosciences) using Image Studio Software 6.0.

IHC slides were scanned using a Leica SCN 400 F Scanner.

Data analysis

Outlines of phase and GFP-positive (where appropriate) acini were generated using a custom pipeline in CellProfiler (Version 3.1.8). A custom macro in ImageJ (2.0.0-rc-9) software was then used to colour code images from each time point, progressively coloured along a blue-to-red rainbow timescale, and concatenate them into one image per 12-hour block to reduce data dimensionality of multiday imaging. Using CellProfiler (Version 3.1.8) measurements such as Area and Compactness, which could reliably measure size and protrusiveness of 3D PC3 objects respectively, were generated for each 12-hour block. Custom pipelines designed in KNIME Data Analytics Platform (Version 3.3.1) were then used to collate data from multiple experiments, normalise to controls, calculate Z-score and perform statistical analysis using one-way ANOVA. Normalised data and p-values are presented as heatmaps generated in PRISM 7 (GraphPad).

Images taken in super resolution mode on the Airyscan microscope were processed using the Zeiss proprietary ZEN 3.2 software,

exported as TIFF files and processed in ImageJ (2.0.0-rc-9).

Opera Phenix™ High Content analysis system and Harmony High-Content Imaging and Analysis Software (PerkinElmer, Version 4.6) was used to perform machine learning. For 2D morphology assays cells were classified as either spindle, spread or round phenotypes using Harmony software (Version 4.6). A custom pipeline was generated using KNIME Data Analytics Platform (Version 3.3.1) to collate data, calculate the log2 fold change of each phenotype over control and to calculate statistical significance using one-way ANOVA. In some cases the intensity of specific proteins in sub-cellular regions such as nucleus, cytoplasm and plasma membrane was also calculated and the data Z-score normalised.

Colocalization between mNeonGreen-tagged ARF5 or ARF6 and RFP-GGA1-NGAT was also quantified using Harmony High-Content Imaging and Analysis Software (Version 4.6) . mNeonGreen and RFP spots were detected and the % green spot area overlapping with red calculated per cell

Phosphoinositide intensity was calculated using an adapted version of PAPI (Pipeline for semi-Automated Phosphoinositide Intensity analysis) described previously. Briefly, cells expressing mNeonGreen-tagged PH domains specific to each PIP were stained for cortex (phalloidin), cytoplasm (CellMask), and nuclei (Hoechst) as described above. Combinations of these dyes were used to create subcellular regions and the relative PIP probe intensity per area per cell measured.

These pipelines were adapted to measure the spot intensity of anti-Akt phosho-S473 in either the juxtanuclear, cytoplasmic or peripheral region of each cell and of anti-Met phospho-Y1234/1235 at the periphery.

Incucyte ZOOM Live Cell Analysis System software (Essen Biosciences, 2018A) was used to analyse the relative wound density of each well (RWD) in migration and invasion assays.

MS data acquisition were performed using the XCalibur4.3 software (Thermo Fisher Scientific). The MaxQuant software version 1.5.5.1 was used to process MS Raw files and searched with Andromeda search engine, querying UniProt.

Primary tumours were imaged and tumour area and volume measured using VevoLAB ultrasound equipment and VevoLAB 3.1.1 software.

Images from IHC were analysed using Halo Image analysis platform (Indica Labs) using CytoNuclear v1.5 algorithm.

cBioPortal.org. tools were used to analyse patient data (copy no., mutational status, RNAseq, RPPA, co-expression matrices and clinical annotation.

qPCR data were analysed using the Applied Biosystems 7500 Software v2.0.6 and the relative quantitation (RQ) was calculated using the comparative Ct (ΔΔCt) method.

For manuscripts utilizing custom algorithms or software that are central to the research but not yet described in published literature, software must be made available to editors/reviewers. We strongly encourage code deposition in a community repository (e.g. GitHub). See the Nature Research guidelines for submitting code & software for further information.

Data

Policy information about availability of data

All manuscripts must include a data availability statement. This statement should provide the following information, where applicable:

- Accession codes, unique identifiers, or web links for publicly available datasets
- A list of figures that have associated raw data
- A description of any restrictions on data availability

The proteomic data generated in this work have been deposited as partial submission to the ProteomeXchange Consortium via the PRIDE partner repository 84 with the dataset identifier PXD013810 [http://www.ebi.ac.uk/pride/archive/projects/PXD013810]. Any other data that supports the findings of this study are available from the corresponding author upon reasonable request.

Uncropped and unprocessed blots are available in Source data file.

The RNAseq data from PC3 sublines in this study are available in either the Short Read Archive database for PC3E versus GS689.Li, SRS354082 [https://www.ncbi.nlm.nih.gov/sra/?term=SRS354082]), or the Gene Expression Omnibus for PC3-Epi versus PC3-EMT14, GSE48230 [https://www.ncbi.nlm.nih.gov/geo/query/acc.cgi?acc=GSE48230].

Normal versus Tumour RNAseq, including transcript variant expression, was obtained by downloading IQSEC1 variant annotation across the TCGA pan cancer dataset using the TCGA Splicing Variants Database (www.TSVdb.com)

Field-specific reporting

Please select the one below that is the best fit for your research. If you are not sure, read the appropriate sections before making your selection.

☒ Life sciences ☐ Behavioural & social sciences ☐ Ecological, evolutionary & environmental sciences

For a reference copy of the document with all sections, see nature.com/documents/nr-reporting-summary-flat.pdf

Life sciences study design

All studies must disclose on these points even when the disclosure is negative.

Sample size	Our studies involve growth of cells as 3D cysts, often in a 96-well plate to allow for high-throughput imaging. Germaine to this is plating of cysts with appropriate density (neither too dense or sparse) to facilitate optimal growth without overcrowding, the latter of which causes aberrant merging of cysts. In this format, we can image hundreds to thousands of spheroids per condition. Such high samples numbers preclude the need to pre-calculate minimal sample size to achieve significant effects, allowing robust statistical depth.
Data exclusions	No data were excluded in analyses.
Replication	Where appropriate number of biological and technical replicates are stated in Figure Legends.
Randomization	Due to the nature of this research, randomization is not appropriate as assignment to categories must be exact. Due to the automated nature of our analysis pipelines users have no control of raw values generated from experiments.
Blinding	Blinding was not performed. However, experimenter bias was circumvented by the use of automated imaging and data analysis pipelines.

Reporting for specific materials, systems and methods

We require information from authors about some types of materials, experimental systems and methods used in many studies. Here, indicate whether each material, system or method listed is relevant to your study. If you are not sure if a list item applies to your research, read the appropriate section before selecting a response.

Materials & experimental systems

n/a	Involved in the study	
☐	☒	Antibodies
☐	☒	Eukaryotic cell lines
☒	☐	Palaeontology
☐	☒	Animals and other organisms
☒	☐	Human research participants
☒	☐	Clinical data

Methods

n/a	Involved in the study	
☒	☐	ChIP-seq
☒	☐	Flow cytometry
☒	☐	MRI-based neuroimaging

Antibodies

Antibodies used	Antibodies used for western blotting were as follows; anti-GAPDH (CST 2118 1:5000), anti-IQSEC1 (Sigma G4798), anti-IQSEC1 (Caltag-Medsystems PSI-8009), anti-GFP (Merck 000000011814460001), anti-LRP1 (Sigma L2295), anti-Met (CST 3127), anti-Met phospho 1234/1235 (CST 3077), anti-Akt (CST 2920), anti-Akt phospho S473 (CST 3787), anti-ARF1 (Novus Biologicals NB-110-85530), anti-ARF6 (Sigma A5230), ARF5 (Novus Biologicals H00000381-M01), anti-ARFGAP1 (Sigma HPA051019), anti-SORL1 (BD 611860), anti-Sin1 (CST 12860), anti-RICTOR (CST 2114), anti-PPC6 (Sigma HPA050940 1:250), anti-14-3-3ζ/Δ (CST 7413), (anti-PIP5Ka (CST 9693) and PIP5Kb (Sigma K0767).<br><br>Antibodies used for IF/IHC; Alexa Fluor 488 (A12379), 568 (A12380) or 647 (A22287) phalloidin (Life Technologies), anti-IQSEC1 (Sigma, G4798), anti-IQSEC1 (Caltag-Medsystems PSI-8009), anti-LRP1 (Sigma L2295), anti-Met (CST 3127), anti-Met phospho 1234/1235 (CST 3077), anti-Akt phospho S473 (CST 3787), anti-Ki67 (Thermo Fisher 18-0192Z), anti-CC3 (CST 9661), anti-Ki67 (Abcam ab16667), anti-PtdIns(3,4,5)P3 and anti-PtdIns(4,5)P2 (Echelon Biosciences, Z-P345B and Z-P045).
Validation	In this work we have validated the following antibodies in western blots using shRNA, overexpression of target proteins or the addition of specific inhibitors: anti-IQSEC1 (Sigma G4798), anti-IQSEC1 (Caltag-Medsystems PSI-8009), anti-LRP1 (Sigma L2295), anti-Met (CST 3127), anti-Met phospho 1234/1235 (CST 3077), anti-Akt (CST 2920), anti-Akt phospho S473 (CST 3787), anti-ARF1 (Novus Biologicals NB-110-85530), anti-ARF6 (Sigma A5230), ARF5 (Novus Biologicals H00000381-M01), anti-ARFGAP1 (Sigma HPA051019), anti-SORL1 (BD 611860), anti-Sin1 (CST 12860), anti-RICTOR (CST 2114), anti-PPC6 (Sigma HPA050940 1:250), anti-14-3-3ζ/Δ (CST 7413), (anti-PIP5Ka (CST 9693) and PIP5Kb (Sigma K0767).<br><br>In addition antibody validation information is available from the manufacturers.

Eukaryotic cell lines

Policy information about cell lines

Cell line source(s)	PC3 (ATCC), PC3 E-Cad+, TEM4-18, TEM2-5, GS689.Li, GS694.LAd, GS683.LALN, JD1203.Lu, GS672.Ug (M. Henry, University of Iowa), PC3-Epi and PC3-EMT (K. Pienta, Johns Hopkins School of Medicine) and 22Rv1 and LNCaP cells (H. Leung, Beatson Institute purchased from ATCC) .

October 2018

	RWPE-1, RWPE-2, WPE-NB14, DU145, VCAP and CA-HPV-10 cell lines (ATCC).<br>HEK293-FT (Thermo Fisher Scientific).<br>KC Ptenfl/+59 (J. Morton, CRUK Beatson Institute)<br>TKCC-07 (Australian Pancreatic Cancer Genome Initiative (APGI) at the Garvan Institute of Medical Research (www.pancreaticcancer.net.au))<br>MDA-MB-231 (ATCC),
Authentication	PC3, LNCAP, DU145, HEK293-FT, RWPE-1 and RWPE-2 cells were authenticated using short tandem repeat (STR) profiling in house (Promega Geneprint 10).
Mycoplasma contamination	We confirm that our cell lines are routinely tested for Mycoplasma, and verify that these are Mycoplasma-free (Mycoalert, Lonza).
Commonly misidentified lines<br>(See ICLAC register)	No commonly misidentified cell lines were used in this study.

Animals and other organisms

Policy information about studies involving animals; ARRIVE guidelines recommended for reporting animal research

Laboratory animals	7-week-old CD1-nude male mice were obtained from Charles River (UK). Mice were housed at ambient temperature (19-22°C), with relative humidity of 45-65%, and a 12h-12h light-dark cycle.
Wild animals	No wild animals were used in this study.
Field-collected samples	No Field-collected samples were used in this study.
Ethics oversight	Animal experiments were performed in compliance with all relevant ethical regulations and approvals of the relevant UK Home Office Project Licence (70/8645) and carried out with ethical approval from the Beatson Institute for Cancer Research and the University of Glasgow under the Animal (Scientific Procedures) Act 1986 and the EU directive 2010, and sanctioned by Local Ethical Review Process (University of Glasgow).

Note that full information on the approval of the study protocol must also be provided in the manuscript.
