# Supplementary material for: An ARF GTPase module promoting invasion and metastasis through regulating phosphoinositide metabolism
Source: Nat Commun. 2021 Mar 12;12:1623. doi: 10.1038/s41467-021-21847-4 (PMC7955138; doi:10.1038/s41467-021-21847-4)

a Figure 1h

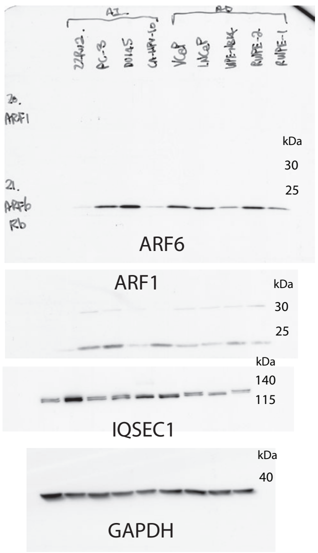

b Figure 2b

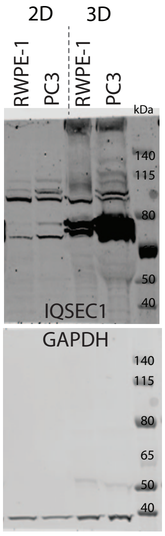

c Figure 2d

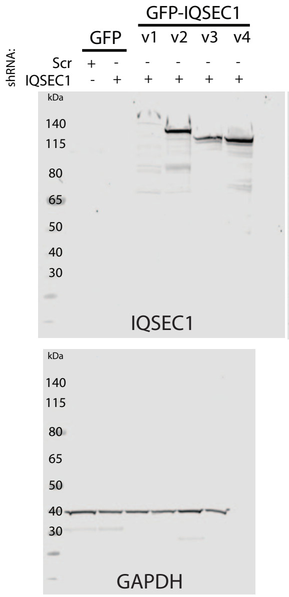

d Figure 2h

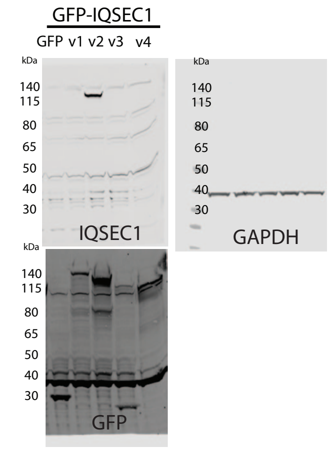

e Figure 3a

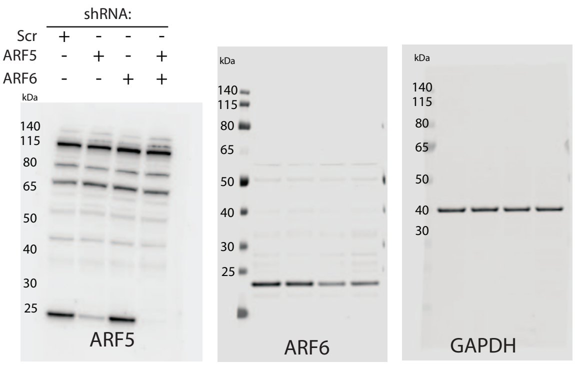

f Figure 3d

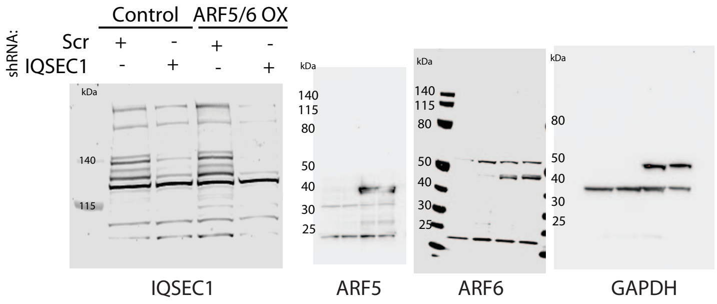

g Figure 4d

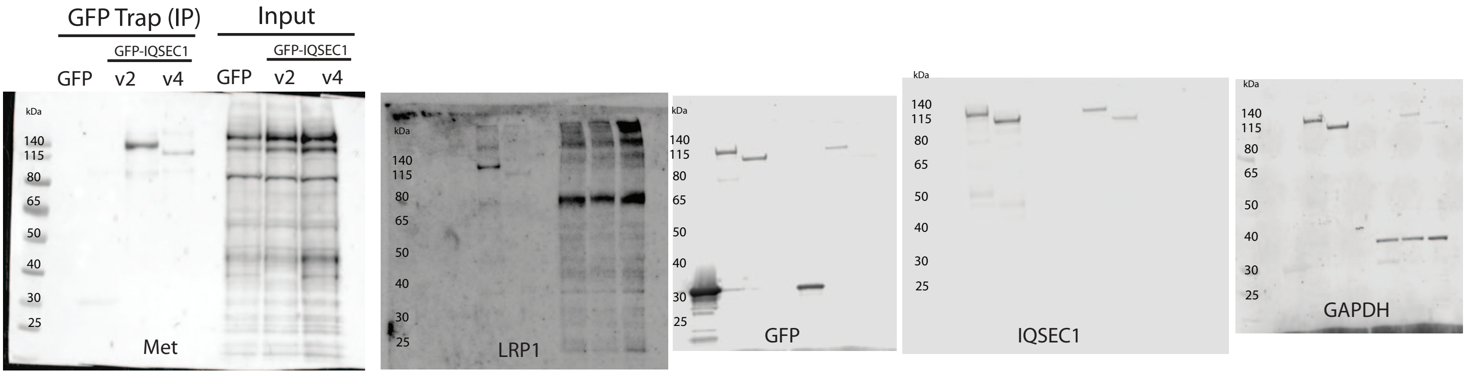

h Figure 5a

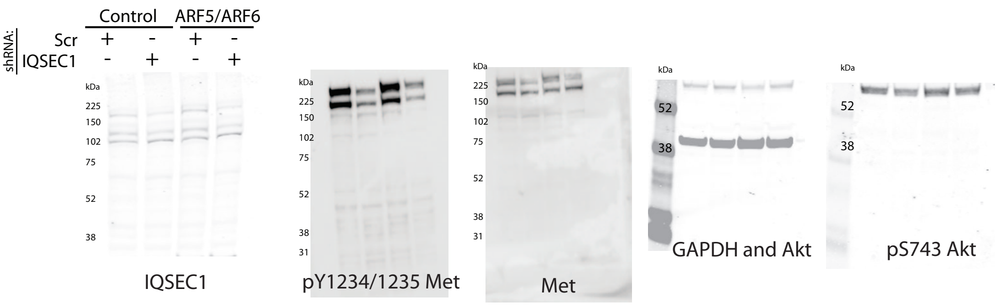

i Figure 6e

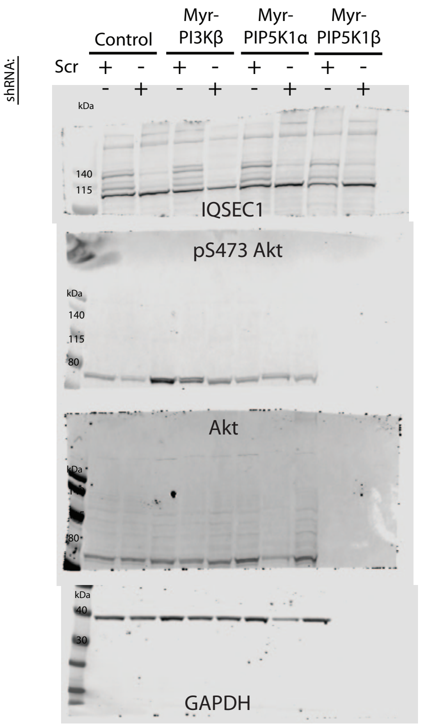

j Figure 6f

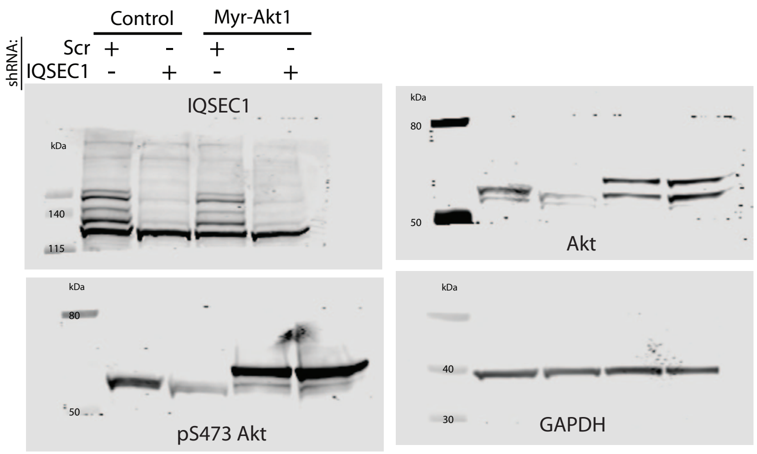

k Supp Fig1a

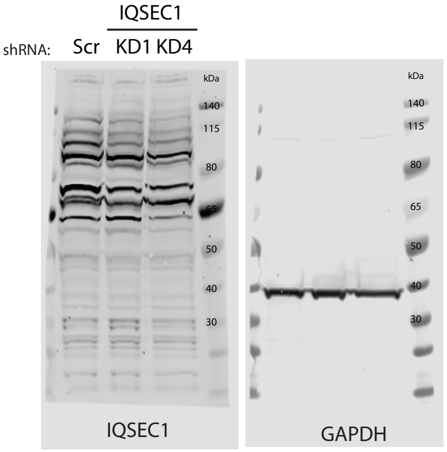

l Supp Fig4c

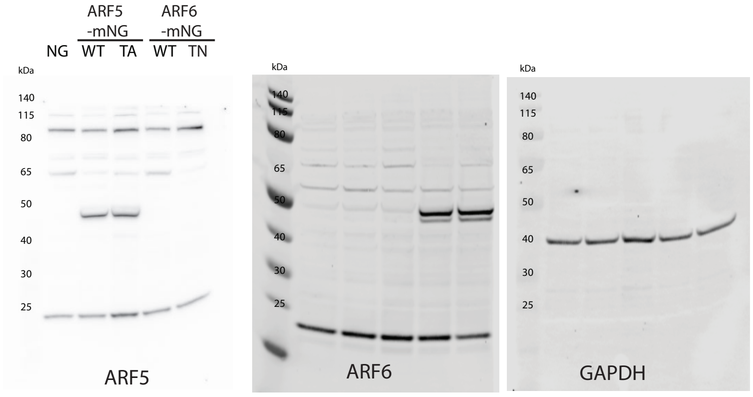

m Supp Fig4i

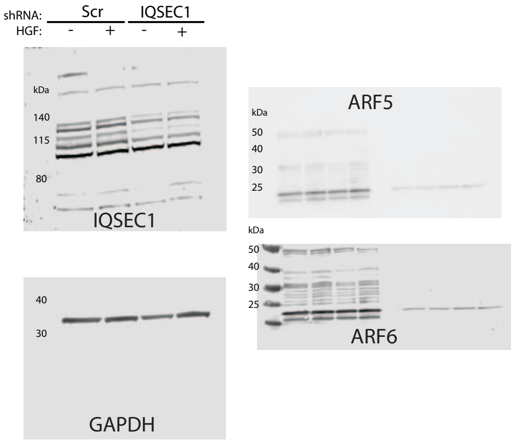

n Supp Fig4l

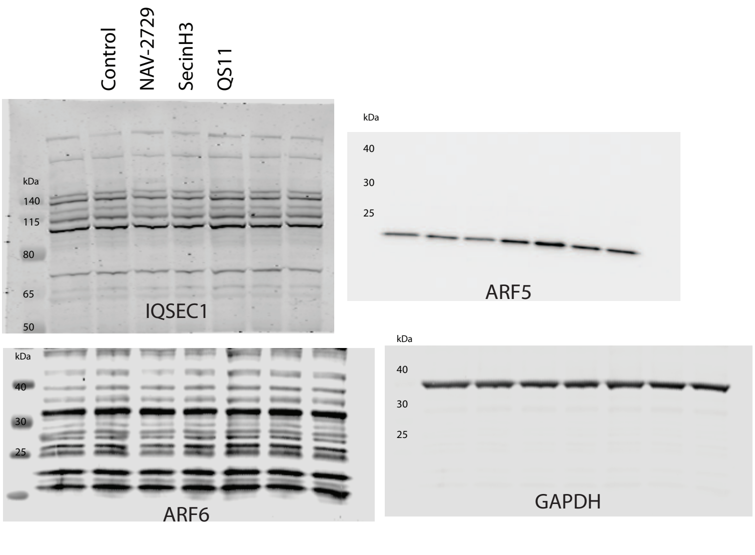

o Supp Fig 5a

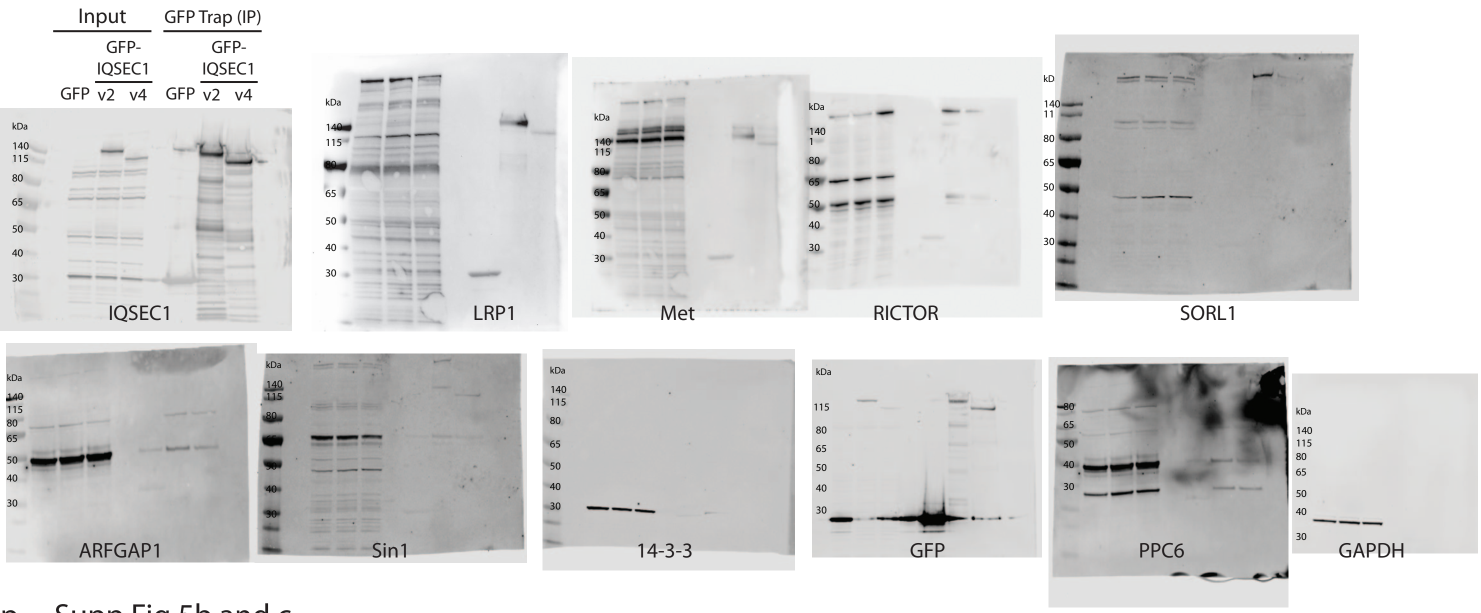

p Supp Fig 5b and c

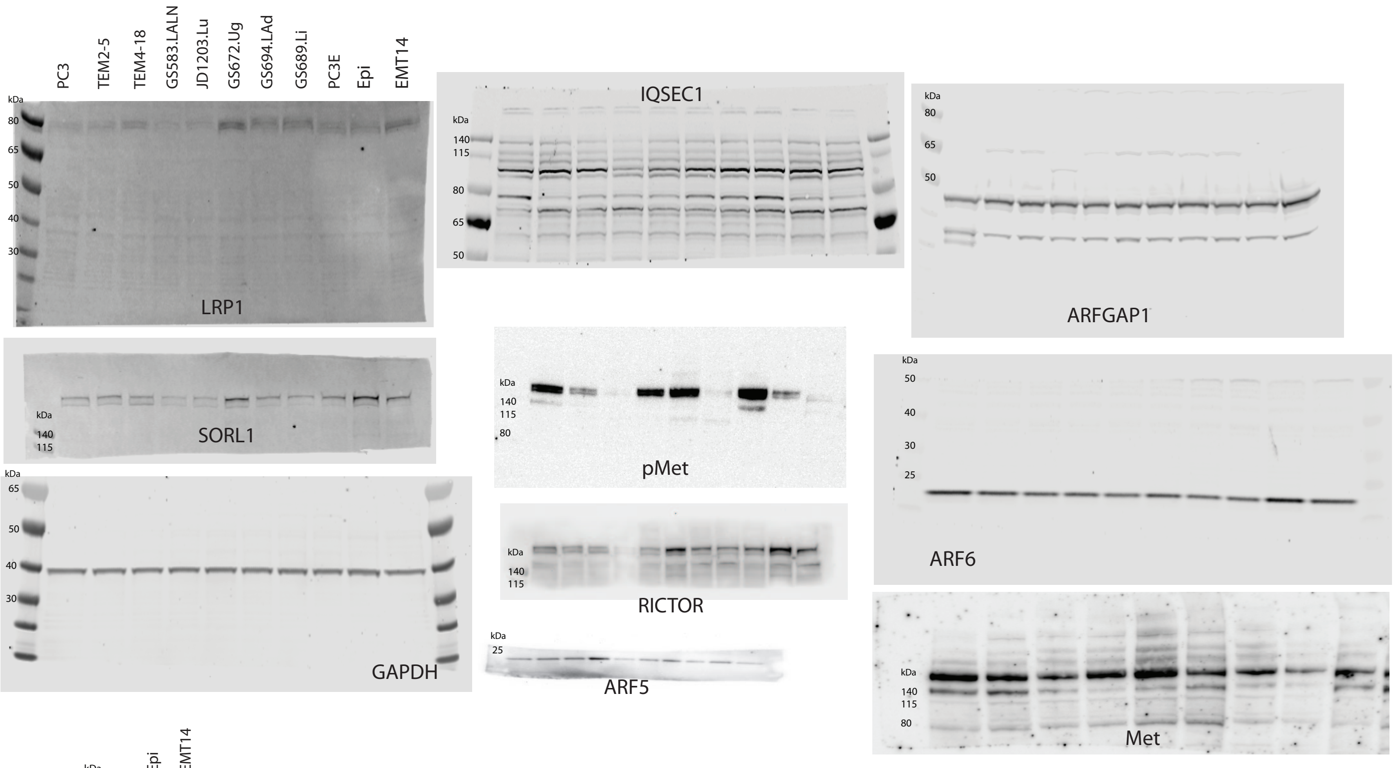

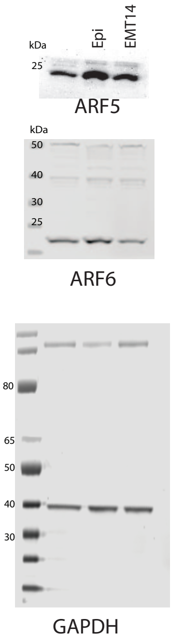

q Supp Fig 5d

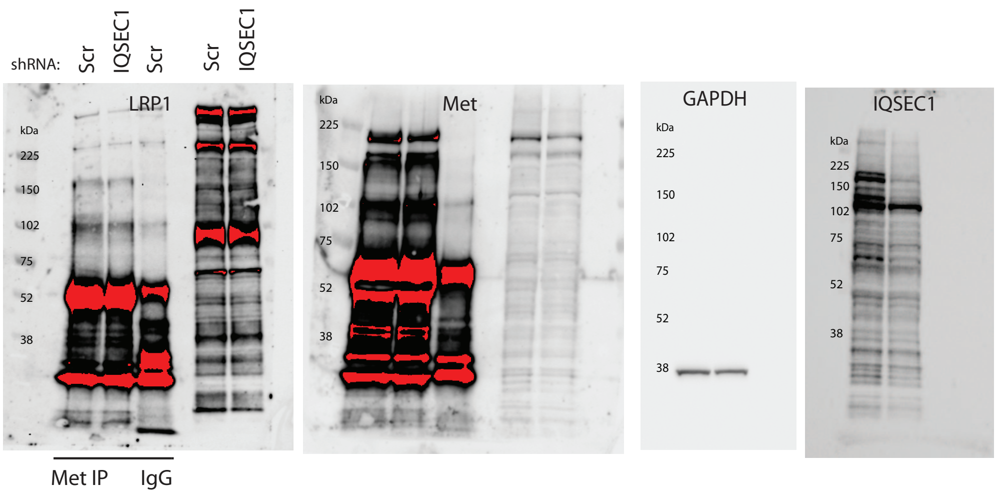

t Supp Fig 6e

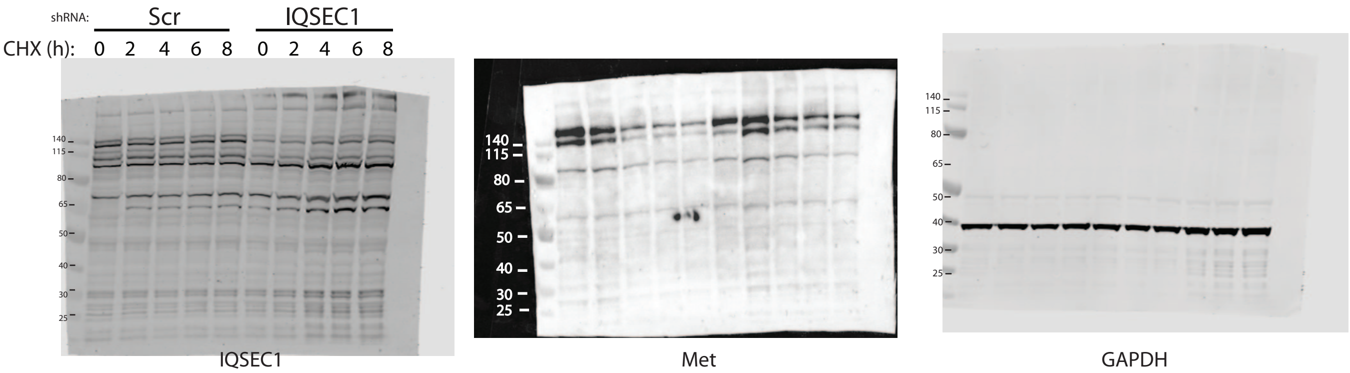

u Supp Fig 8a

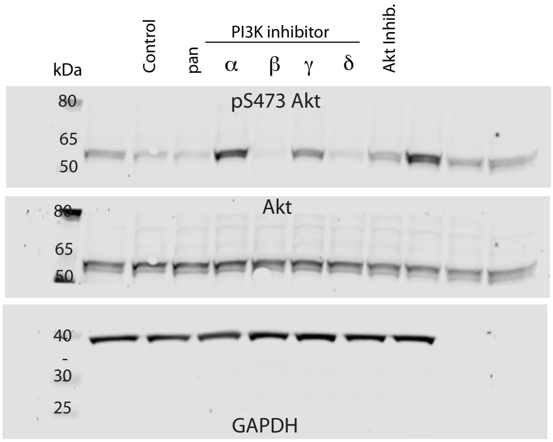

v Supp Fig 8e

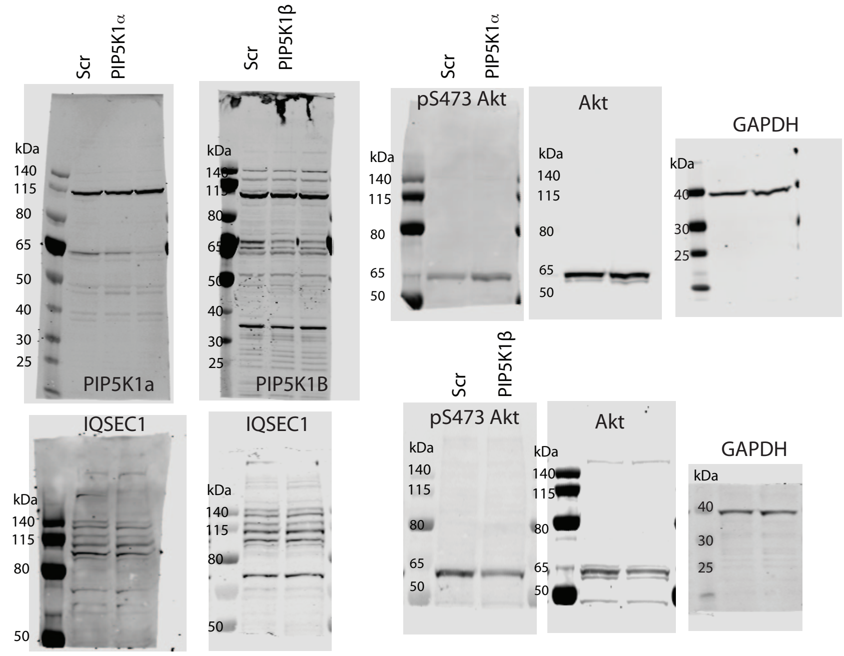

w Supp Fig 9b

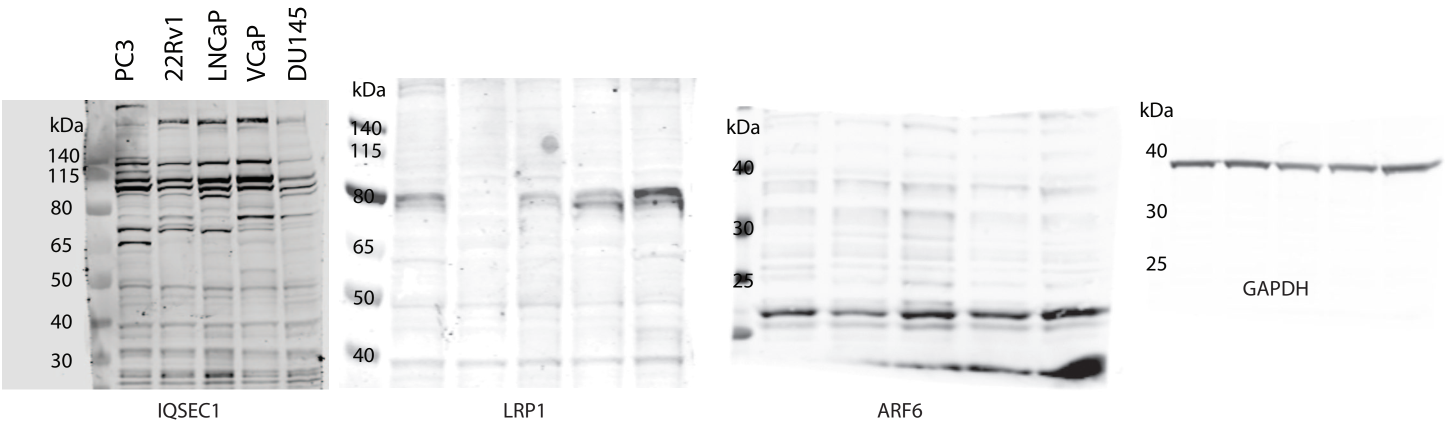

r Supp Fig 5e-f, i, l-n

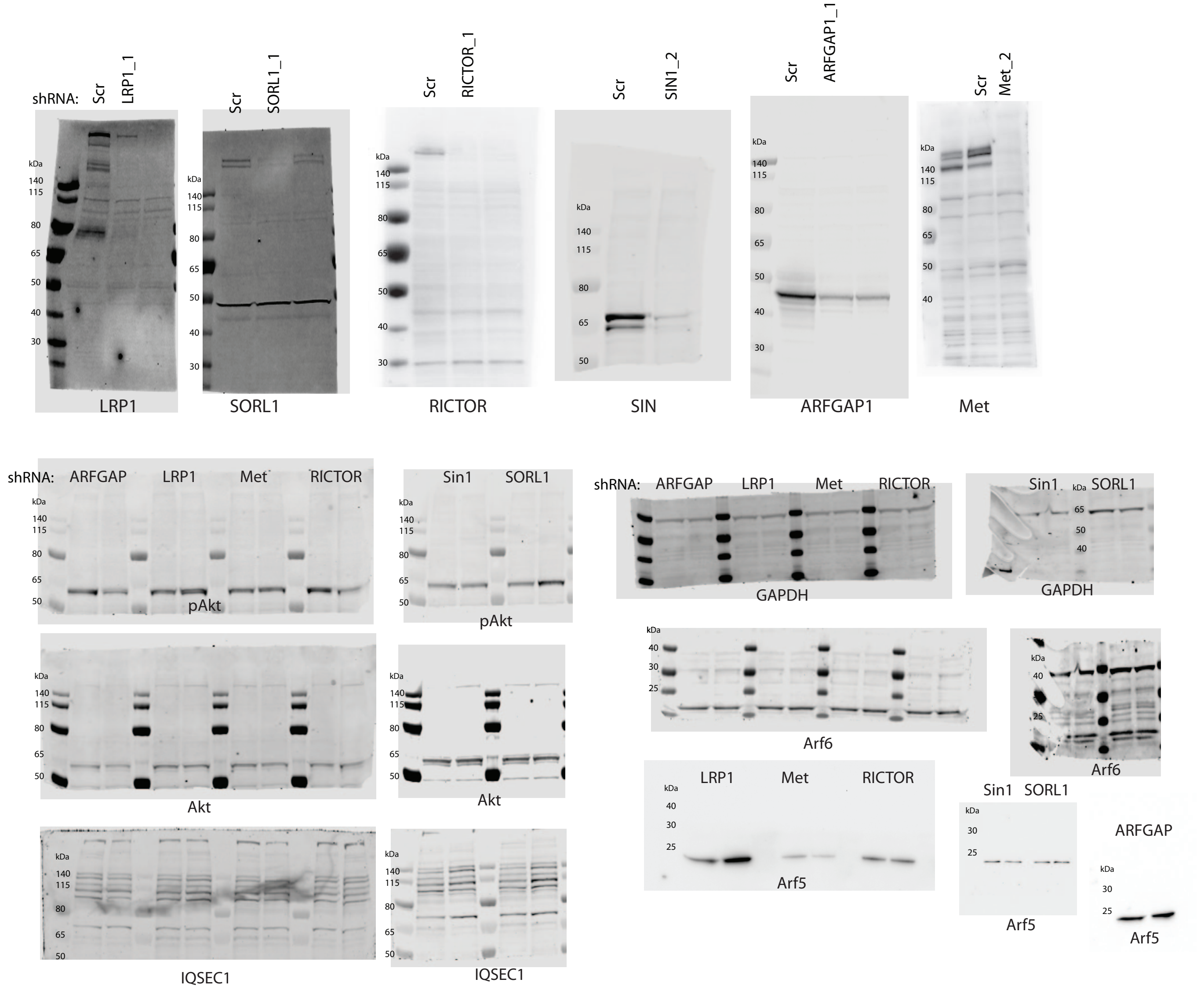

s Supp Fig 6a

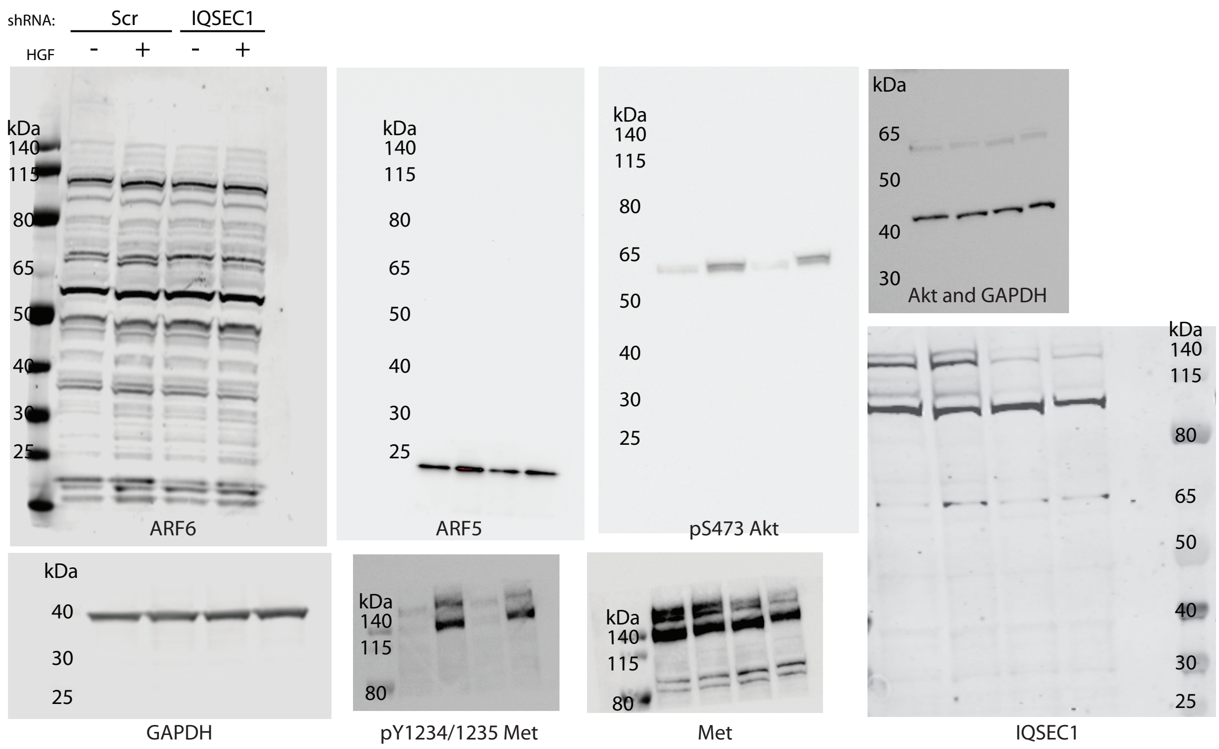

Supplement: Supplementary file 7 — Source Data [file 41467_2021_21847_MOESM7_ESM.pdf]
